# Asilomar Across the Atlantic: EMBO, EMBL, and the Politics of Scientific Expertise

**DOI:** 10.1007/s10739-025-09808-9

**Published:** 2025-03-04

**Authors:** Francesco Cassata, Soraya de Chadarevian

**Affiliations:** 1 University of Genoa, Genoa, Italy; 2 Centro Linceo Beniamino Segre, Rome, Italy; 3 University of California, Los Angeles, Los Angeles, USA

**Keywords:** Recombinant DNA, Asilomar, EMBO, EMBL, Science policy

## Abstract

The internationalization of the 1975 International Asilomar Conference on Recombinant DNA molecules has received little attention, and in particular, the European impact on, and response to, the Asilomar Conference have remained largely unexplored in the historiography to date. This article highlights the role of the European Molecular Biology Organization (EMBO) as a key actor in recombinant DNA research and the issuing of guidelines for recombinant DNA technology on both sides of the Atlantic. It also investigates the legacy of the Asilomar Conference in shaping EMBO’s role as a science policy advisor for molecular biology in Europe. Drawing on a wide range of primary sources, the article is divided into three sections. The first section explores EMBO’s role as a scientific advisory body in the development and guidance of recombinant DNA research in both the US and Western Europe. The second section investigates the impact of the Asilomar Conference on the European Molecular Biology Laboratory (EMBL) project, reconstructing the scientific and political rationale behind the early construction of a high-risk containment facility in Heidelberg (soon obsolete due to the international relaxation of the guidelines). The third and final section analyzes how, between 1975 and 2004, EMBO reframed the Asilomar legacy as a model for its aspirations to serve as an advisory group for European science policy in molecular biology.

## Introduction

The International Conference on Recombinant DNA molecules, held under the auspices of the US National Academy of Sciences at the Asilomar Conference Center in Pacific Grove, California in February 1975 (hereafter, Asilomar Conference), was convened to review the rapid advancements in recombinant DNA (rDNA) research and to discuss approaches that minimized potential biohazards stemming from rDNA molecules. As a policy-oriented meeting, the 1975 Asilomar Conference was conceived from the outset as an international event. Among the participants, almost one-third came from outside the United States. The European contingent consisted of thirty-five scientists, more than half of whom were members of the European Molecular Biology Organization (EMBO), which had a hand in the organization of the meeting. The Conference outlined a set of safety procedures to enable the resumption of rDNA research, following a voluntary world-wide deferral of certain kinds of experiments called for in a letter published in *Science* and *Nature* in July 1974 by a group of concerned scientists, headed by Stanford biochemist Paul Berg and sponsored by the US National Academy of Sciences.

In the post-Asilomar debate over the regulation of rDNA research, several international organizations besides EMBO quickly became involved. These included United Nations agencies like the World Health Organization (WHO); international non-governmental organizations of regional and global breadth, such as the European Science Foundation (ESF) and the International Council of Scientific Unions (ICSU), respectively; and intergovernmental organizations like the European Economic Community (EEC).

Despite this, the internationalization of Asilomar has hitherto received little attention in the historiography. Classical works on the topic (Wright 1994; Gottweis 1998) have compared the US debate with three other national contexts (UK, France, and Germany), but only briefly addressed the supranational and transnational dimensions.^1^ With a few notable exceptions (Cantley 1995; Gottweis 2005; Jasanoff 2005), the European impact on, and response to, the Asilomar Conference and the subsequent history of the rDNA guidelines has remained largely unexplored.^2^

To shift the focus away from a US-centric analysis of the Asilomar Conference and emphasize its broader international impact, this article examines the European side, particularly EMBO’s role as a fundamental transnational actor in rDNA research and its regulation.

Two reasons justify the decision to highlight EMBO among other international organizations: the first is chronological, the second is conceptual. Firstly, EMBO had a major role in the immediate internationalization of the Asilomar Conference: it co-sponsored the meeting, and the one European on the organizing committee, Cambridge molecular biologist Sydney Brenner, was an active member of EMBO. Secondly, and more importantly, the unique institutional framework of molecular biology in Europe is crucial for understanding the close scientific, political, and technological interactions and exchanges between the United States and Western Europe in the field of rDNA research. A complex, three-dimensional architecture emerged in European molecular biology during the 1960–1970s, including a private scientific association (EMBO), an intergovernmental agency (the European Molecular Biology Conference, EMBC), and a central laboratory of molecular biology (the European Molecular Biology Laboratory, EMBL). Through the lens of the EMBO/EMBC/EMBL framework, the impact of the Asilomar Conference in Western Europe becomes more significant, encompassing not only scientific networks but also intergovernmental relationships and technological infrastructures.

Founded in 1964 as a private association with headquarters in Geneva, EMBO functioned as a club of self-appointed elite scientists committed to a dual-purpose mission: firstly, organizing an intra-European networking system for competitive research funding, encompassing fellowships, travel grants, advanced courses, and workshops; secondly, establishing a central European laboratory of molecular biology. These two complementary projects were meant to counter the so-called American challenge and brain drain from Western Europe to the United States (de Chadarevian 2002; Krige 2002; Strasser 2003; Cassata 2015).

In February 1969, the signing of an international treaty by twelve Western European countries marked the birth of the EMBC.^3^ This intergovernmental structure ensured political legitimacy and permanent public funding for EMBO activities. The treaty defined a special relationship between EMBO and EMBC. Initially for a five-year period (extended to eight years after 1980), the EMBC approved the scientific program outlined by EMBO, including fellowships, courses, workshops, lectureships, and visiting professorships. Crucially, the EMBC entrusted EMBO with the exclusive administration of this program, creating the unique situation in which governmental funds were transferred from an intergovernmental organization to a purely private international association of scientists (Cassata 2024). Furthermore, the EMBC ensured funding for the planning of EMBL as an EMBC Special Project. The construction of this unique tripartite system owed much to the science policy and diplomatic skills of John Kendrew, who for a long time single-mindedly pursued the project of building a central European laboratory for molecular biology research with state-of-the-art facilities. This vision would reach fruition in the EMBL, legally established in 1974, and its new facilities were inaugurated in 1978 in Heidelberg.

The 1975 Asilomar Conference occurred at a critical moment in the definition of this scientific, political, and institutional framework. Between 1973 and 1978, EMBO had to convince EMBC member states to renew their funding agreement, respectively for the periods 1975–1980 and 1980–1988. Concurrently, between 1974 and 1978, the EMBL was under construction, and its scientific program was not fully implemented. In this fluid context, the Asilomar Conference not only sparked new interest in rDNA research but also became a scientific and political resource to be mobilized in the effort to define molecular biology in Western Europe.

Established in the early 1960s to close the perceived gap between Western Europe and the United States, EMBO found itself faced with the same challenge a decade later. The American call for a temporary suspension of certain rDNA experiments in July 1974 caused alarm within EMBO circles as scientists feared this decision could worsen Europe’s lag in the rapidly advancing field of rDNA research. In his interview with MIT historian of science Charles Weiner, in May 1975, Charles Weissmann, EMBO Council member and director of the Zürich Institute for Molecular Biology, clearly articulated the European concerns at the time:

One person concretely said to me that the Americans were setting up a situation of a closed shop, where they had set up the system, gotten out some very interesting first results and they would probably continue exploring them while in fact they were propagating a moratorium, and prevent this technique from being taken up by others. That was stated, rightly or wrongly; but certainly the idea was around.^4^

In the aftermath of Asilomar, EMBO had the opportunity to demonstrate the importance of its mission by promoting training and research in rDNA and by providing European researchers with safe laboratory infrastructures at EMBL, in Heidelberg. In a March 1976 interview, EMBO Executive Secretary John Tooze expressed this pursuit of opportunity in bold terms: “So professionally EMBO says, right, we must make sure that though Stanford be Mecca, somewhere in Europe ought to be at least Jerusalem and it’s our responsibility to do that.”^5^

However, to provide the European “Jerusalem” of rDNA research to the American “Mecca,” EMBO had to fully leverage its political and intergovernmental channels within the EMBC context. This involved developing its role as an expert body and acting as a transnational science policy advisor. On the one hand, EMBO needed to align guidelines between the United States and Europe to avoid competitive disadvantages. To achieve this, EMBO had a significant impact on the definition of the US guidelines and their subsequent relaxation, while at the same time advocating for their consistent application across Western Europe. On the other hand, it had to oversee the political and administrative implementation of these guidelines in Western Europe to facilitate intra-European research and exchanges. This required EMBO to navigate the delicate balance between technical expertise and political advice in a landscape of growing competition with Western European government research agencies and other European organizations, such as the ESF, a nongovernmental organization established in 1974 outside the European Community structures and led by national research councils and academies.

The following section examines EMBO’s role as a scientific advisory body for the development of guidelines for rDNA research in both the US and Western Europe. In particular, it highlights how through the Asilomar Conference, the EMBO Standing Advisory Committee on rDNA (hereafter, EMBO SAC) influenced the initial shaping and the subsequent dismantling of the US guidelines, while concurrently working toward harmonizing regulatory frameworks in Western Europe and aligning them with those of the United States. The second section investigates how the Asilomar Conference shaped the subsequent design and research policies of the EMBL, while the third section analyzes how for three decades, from the mid-1970s to the early 2000s, the experience of the EMBO SAC was framed as a model for EMBO’s broader science policy aspirations.^6^

## Framing Guidelines for the United States and Western Europe: The EMBO Standing Advisory Committee on Recombinant DNA (1975–1980)

Potential risks related to the emerging technology of rDNA were discussed in EMBO circles well before the Gordon Research Conference on Nucleic Acids in June 1973 that is often cited as the event that brought the issue to broader awareness. In July–August 1971, EMBO Council member Hans Georg Zachau of the University of Munich organized an EMBO-NATO Summer School on Molecular and Developmental Biology at Erice, on the hills of Western Sicily. Stanford biochemist Paul Berg, a recent recipient of a short-term EMBO fellowship to work on cancer viruses at the Imperial Cancer Research Fund (ICRF) in London, gave a lecture on his research, focusing on the construction of circular hybrid molecules containing DNA from lambda phage and from the monkey tumor virus SV40. A special evening session was dedicated in Erice to discussing the political and social implications of genetic engineering. “We sat up till about midnight,” Berg recollected, “this whole crew drinking beer, about eighty people, back and forth discussing the possible hazards of and prospects for genetic engineering.”^7^

Later Zachau recalled Berg’s talk as a crucial moment of awareness:

He talked there, among other things, about the experiments which in 1972 appeared in the *PNAS* [*Proceedings of the National Academy of Sciences*], and which were the beginning of all the concern at the Gordon Conference, and subsequently Asilomar, and so on. That was the first time I was formally introduced into [*sic*] the experimental design.^8^

One year later, at the end of September 1972, about fifty molecular biologists from twelve  countries, including a score from the United States, attended the EMBO Workshop on DNA Restriction and Modification. The event, organized by Swiss microbiologist Werner Arber, was held near Basel, at the conference center of Leuenberg, renowned since 1969 as a venue for interconfessional dialogues among European Protestant churches. On this occasion, new information about restriction enzymes producing staggered, or sticky, ends at cleavage sites in DNA, and the implications for novel genetic manipulation, were discussed (Loenen 2014, p. 3). One evening of the workshop was devoted to “an open discussion of the use of restriction endonuclease to construct genetic hybrids between DNA molecules and the implications this may have as a useful tool in genetic engineering and the potential biohazards” (Fredrickson 1991, p. 269).

The alarm bell rang again in June 1973, this time reaching the pages of *Science.* At the Gordon Research Conference on Nucleic Acids, held in New Hampshire, Herbert Boyer shared information about the capabilities of the restriction enzyme EcoRI to splice DNAs of different origin and how two plasmids bearing genes specifying resistance to two different antibiotics had been joined. His talk was received with enthusiasm, but it also sparked concerns about potential biohazards. At the request of the graduate students at the conference, the meeting co-chairpersons, NIH biochemist Maxine Singer,^9^ and Yale researcher Dieter Söll,^10^ wrote a letter to the US National Academy of Sciences (NAS).^11^ In this letter, they warned about the creation of “new kinds of hybrid plasmids or viruses, with biological activity of unpredictable nature” that “may prove hazardous to laboratory workers and to the public” (Singer and Söll 1973). They also called on the US NAS and its health branch, the US Institute of Medicine (IOM), to set up a study group to investigate the problem and develop guidelines for action. The letter appeared in *Science* in September 1973, soon attracting international attention.

Reacting to the Singer-Söll letter, the US NAS asked Berg, as an Academy member and a key figure in the field, to establish a committee to examine the problem and to propose short- and long-term actions. Berg invited a number of leading molecular biologists and biochemists—among them David Baltimore, Herbert Boyer, and Stanley Cohen, who were centrally involved in the development of the new techniques—to prepare an open letter to the scientific community containing a series of proposals to deal with the rDNA issue.^12^ The final version of this document (known as the Berg letter), published in July 1974 in the *Proceedings of the National Academy of Sciences*, in *Nature*, and in *Science* (Berg et al. 1974), included four recommendations. First, until the potential hazards of rDNA molecules have been “better evaluated,” the signatories and “scientists throughout the world” should voluntarily defer two types of experiments, those involving plasmids that might confer novel resistances to antibiotics or the ability to make toxins upon bacteria, and those involving the introduction of oncogenic or other animal viruses into plasmid or phage DNA that could infect bacteria. Second, researchers had to “carefully weigh” experiments linking animal DNA to plasmid or phage DNA. Third, the National Institutes of Health (NIH)—the primary federal funder of biomedical and public health research in the US—should establish an advisory committee to oversee an experimental program of research exploring the hazards of rDNA technology, to develop procedures for minimizing the spread of rDNA molecules, and to draw up guidelines for work in this field; finally, an international meeting involving scientists from all over the world should be convened to review scientific progress in this area and to discuss appropriate ways to deal with the potential biohazards of rDNA molecules (Campos 2024).

Prior to its publication, the contents of the Berg letter circulated among EMBO members through international meetings. On May 20–24, 1974, at the EMBO Workshop on Restriction Enzymes and DNA sequencing held in Ghent, Belgium, Johns Hopkins microbiologist Daniel Nathans anticipated the recommendations of the Berg letter, receiving general agreement from the audience.^13^ A few days later, on the other side of the Atlantic, at the Cold Spring Harbor Tumor Viruses Symposium (May 30–June 6, 1974), it was the turn of David Baltimore to disseminate the contents of the forthcoming Berg letter.

Among the attendees at Cold Spring Harbor were Charles Weissmann and the Swedish virologist Lennart Philipson, both EMBO Council members and both prospective users of rDNA technologies. They promptly drafted a letter to EMBO Secretary General John Kendrew. The letter was signed by ten other researchers, all EMBO members, working in Western Europe and Israel. In view of the incoming publication of the Berg letter, Weissmann and Philipson recommended that “the problems” related to rDNA technology “be urgently and carefully considered by appropriate EMBO bodies, both in regard to security recommendations and to providing appropriate special risk laboratories within the framework of EMBO, for instance at the Heidelberg Laboratory.”^14^ Although Kendrew acknowledged that “some kind of standing committee was needed to monitor the biological hazards,” his initial reaction to Weissmann’s and Philipson’s suggestions was rather slow and non-committal.^15^

The publication of the Berg letter, followed by the establishment, on July 27, of the first European committee of experts—the UK Working Party on the Experimental Manipulation of the Genetic Composition of Microorganisms, briefly known as the Ashby Committee (from the name of its chairman, the former Cambridge vice-chancellor Eric Ashby)—changed the situation. The Ashby Committee not only included several EMBO members,^16^ but it also asked other EMBO members, namely Sydney Brenner, to provide written testimonies about potential benefits and hazards of the new technology.

On July 19, after consultation with the EMBO Council Chairman, Niels Jerne, and other Council members, Kendrew contacted Berg, expressing support and interest in the work of his committee. Kendrew especially endorsed the proposal to convene an international conference, declaring that EMBO would be glad to be associated with the event and even to collaborate in the organization.^17^ At the beginning of September, Berg visited Cambridge, talked on the BBC *Controversy* program about what the broadcaster called the “world ban of certain genetic experiments,” and met Kendrew at dinner, discussing EMBO’s concrete participation in funding the travel expenses of the Western European researchers attending the forthcoming Asilomar Conference.^18^ On his return to Stanford, Berg invited Sydney Brenner and Niels Jerne to join the Organizing Committee of the Asilomar Conference. In his letter, Berg recognized that he was “desperately” in need of a few more members “particularly from Europe.” “If the Conference is to be international in its representation and impact,” he argued, “non-Americans must be involved in both organizing the meeting and, more importantly, in generating the recommendations that come from the Conference.”^19^ Jerne played no active role and eventually resigned from the committee, possibly disagreeing with the initiative.^20^ Brenner not only accepted Berg’s invitation but suggested contacting Weissmann and the “Edinburgh group,” particularly Ken Murray, who had already introduced mammalian DNA into the lambda phage. Furthermore, as a member of the Ashby Committee, Brenner frequently updated Berg about the development of the situation in the UK. The anticipations of the Ashby report (published in January 1975) sounded optimistic: given appropriate measures of physical and biological containment, potential hazards of rDNA could be kept under control.^21^

By November 1974 Berg had produced a list of potential Western European invitees to the meeting in Asilomar that he shared with Tooze, inquiring how many of these could be supported by EMBO travel grants. By the end of the year, the EMBC decided the allocation of 5000 EUA (around US$6000) “to cover the expenses of the EMBO to an international symposium on genetic engineering in California” (see Table 1).^22^ As Kendrew pointed out in his interview with Charles Weiner, this decision was important not only in economic but also in political terms as it marked the very first involvement of Western European governments in the Asilomar Conference.^23^

**Table 1:** Chronology of events detailing the close interactions between EMBO/EMBC and its American counterparts in the organization of the Asilomar Conference and the subsequent discussions around the NIH guidelines

July 1974	Berg Letter (*Science* 185: 303)
Dec 17, 1974	EMBC allocation of resources for the participation of EMBO representatives in the Asilomar Conference
Jan 10, 1975	Appointment of the EMBO delegation to the Asilomar Conference
Jan 10, 1975	EMBO “ad hoc Committee on Gene Transplantation”
Feb 27, 1975	Report of EMBO “ad hoc Committee on Recombinant DNA Molecules” from the Asilomar Conference to the EMBO Council
July 3, 1975	Official report of the EMBO Asilomar delegation to EMBC governments
Jan 17, 1976	Establishment of the EMBO SAC
Feb 14–15, 1976	1st EMBO SAC meeting (Post House Hotel, Heathrow Airport, London) Discussion of the Ashby Report, the provisional report of the Asilomar Conference, and the proposed La Jolla NIH guidelines Proposal to adopt the NIH guidelines as regulatory framework for Western Europe
Sep 18–19, 1976	2nd EMBO SAC meeting (Post House Hotel, Heathrow Airport, London) EMBO definition of recombinant DNA research Comparison of the NIH guidelines and Williams Report in UK Discussion of the protocol of the EMBO Risk Testing Experiment
March 21–23, 1977	NIH/EMBO Workshop on Parameters of Physical Containment (Ariel Hotel, Heathrow Airport, London)
May 19, 1977	3rd EMBO SAC meeting (IRBM, Paris) Weissmann agrees to act as chair until Spring 1978 Discussion of Canadian, French, and German guidelines Progress report on the EMBO Risk Testing Experiment
Nov 26–27, 1977	4th EMBO SAC Meeting (Post House Hotel, Heathrow Airport, London) Report on the EMBO Risk Testing Experiment Comments on the proposed revision of the NIH guidelines Discussion of French and German draft guidelines Advice to the ESF and the Swedish National Committee
Jan, 27–29, 1978	NIH-EMBO Workshop in Ascot, Berkshire, to Assess Risks for Recombinant DNA Experiments Involving the Genomes of Animal, Plant, and Insect Viruses
Dec 2–3 1978	5th EMBO SAC meeting (Post House Hotel, Heathrow Airport, London) Discussion of NIH revised guidelines and the GMAG’s proposal in UK Report on the EMBO Risk Testing Experiment
Feb 17, 1980	6th EMBO SAC meeting (Geneva) Comments on the revised NIH guidelines (published in 1979) and on the second report of GMAG. Discussion of the regulatory framework in France, West Germany, Netherlands, Sweden. Report on the results of the EMBO Risk Testing Experiment

At their meeting on January 10, 1975, the EMBO Council decided that Walter Bodmer, Philipson, Tooze, Weissmann, and Zachau would attend “Professor P. Berg’s Conference” and report back to the EMBC.^24^ An “ad hoc Committee on Gene Transplantation” was also established “to study the impact of the possible hazards of working with hybrid DNA molecules on the working conditions in European laboratories, and to consider possible precautions which need to be taken.” In addition to the five EMBO delegates to Asilomar, this committee included Brenner, Murray, and the head of the Biochemistry Unity at the Pasteur Institute in Paris (and EMBO Council member) François Gros.^25^

Except for Gros, the whole group attended the Asilomar Conference. On the last day of the meeting, Tooze and some other EMBO delegates, while sitting “on one of those benches by the sea,”^26^ wrote a first report to the EMBO Council.^27^ In July 1975, the EMBO delegation presented an official report on the Conference to the EMBC governments, recommending, first, that the Ashby Report and the Report of the Asilomar Conference be used as interim guidelines for European scientists experimenting with rDNA, and, second, that EMBO establish a Standing Advisory Committee (SAC) on Recombinant DNA Molecules, for the “elaboration, co-ordination, surveillance and review of safety precautions” in this research area in Western Europe, and for cooperation with other international organizations.^28^

The EMBC was asked to take note of the report. Most delegates, notably those from West Germany, France, Sweden, and Switzerland, informed that their own countries had already established—or were considering establishing—national committees dealing with the ethical and technical issues of rDNA technology.^29^ A few, including the delegates from Italy, Austria, Denmark, and Norway, stated that the establishment of national committees was not yet envisaged in their countries. Setting the tone for the discussion, the UK delegation endorsed the establishment of the EMBO SAC, “provided that Committee was given detailed terms of reference including instructions to co-operate with national machinery for the carrying out of that type of work.”^30^ It was particularly emphasized that the proposed EMBO SAC should concern itself “only with the operational and technical aspects of the subject, leaving the regulatory or legislative aspects in the hands of national Governments.” Furthermore, the SAC’s terms of reference should provide for “close liaison between it and the study group being set up within the ESF to investigate the wider implications of work on recombinant DNA molecules, including social responsibility and ethical aspects.”^31^ In the end, the EMBC unanimously supported the establishment of the EMBO SAC, by allocating the necessary resources.^32^

The composition and draft terms of reference for the EMBO SAC were presented at the EMBC meeting of November 1975. Regarding membership, the only addition to the initial group of Asilomar attendees was the British bacteriologist Ephraim S. Anderson. Although he was not yet an EMBO member at the time, his expertise in plasmids and the epidemiology of intestinal infections soon became instrumental in organizing EMBO training courses and risk testing experiments in the field of rDNA.^33^ The draft responsibilities of the SAC were, on request, to advise governments, other organizations and individual scientists about technical and scientific aspects of experiments with rDNA; to arrange training programs on the new technology; to collect copies of laws, rules and guidelines from various countries; and, finally, to maintain close liaison with the ESF and other international governmental and non-governmental organizations that were concerned with the various aspects of rDNA experiments. As the statutes made clear, the EMBO SAC had no regulatory or legislative functions and instead was to concern itself exclusively with scientific and technical questions.^34^

The EMBC took note of these terms of reference, which were approved at the 25th EMBO Council meeting of 17 January 1976. The Council pointed out that the EMBO SAC had to take “a very active role in the discussion of guidelines and in particular consider the relevance of US guidelines to genetic engineering research in Europe.”^35^

Two members of the EMBO SAC, Tooze and Brenner, closely followed the meetings of the NIH Recombinant DNA Advisory Committee (RAC) held in 1975 to draft the guidelines for rDNA research in the United States. In particular, they attended the final meeting in La Jolla, California, in December 1975, where a set of rDNA experiments with correlated levels of physical and biological containment was defined. Upon returning to Heidelberg, Tooze circulated a summary of the La Jolla version of the NIH guidelines to all members of the EMBO SAC, calling for an urgent meeting: “Many of the smaller European countries that are represented in the European Molecular Biology Conference, are expecting the EMBO Committee to give advice on the suitability of the American guidelines for recombinant DNA research in Europe,” he argued.^36^

At its first meeting, at the Post House Hotel of the Heathrow Airport on February 14–15, 1976, the EMBO SAC proposed adopting the forthcoming NIH guidelines as the regulatory framework for rDNA research in Western Europe. Given that attempts to establish risks associated with rDNA research were based more on conjecture than on solid evidence, there was no need—the EMBO SAC argued—to draft a new, specifically *European*, set of guidelines. Moreover, the NIH guidelines had the advantage of internal consistency, even though they were lacking in terms of implementation. The NIH guidelines could thus serve as “a basis for an international code of practice,” at any rate for Western Europe (other than Great Britain, which was implementing its own scheme).^37^ The draft report of the meeting, in a passage omitted in the final version, offers a glimpse into the tone of the discussion:

Throughout the discussion Zachau pointed out that he did not believe the European countries could arrive at a set of guidelines *substantially* different from those applied in the USA. He argued persuasively that the best course of action, indeed the only possible one, was to recommend the adoption, at least in principle, of the American guidelines in Europe, although perhaps some changes in the envisaged implementation would be needed.^38^

In this context, the EMBO SAC outlined a specific and autonomous strategy consisting of two interrelated actions. First, it implemented a range of experiments designed to objectively assess the “putative risks” of rDNA research.^39^ The most significant among these experiments involved measuring “the pathogenicity of polyoma virus DNA attached to a plasmid vector and within *Escherichia coli* when introduced into mice.”^40^ This experimental approach was conceived not only to identify real risks but also to reduce regulatory constraints. Indeed, according to the minutes of that February 1976 EMBO SAC meeting, due to the lack of evidence regarding the potential hazards of rDNA technology, the NIH guidelines should be viewed as “the upper limit of stringency necessary.”^41^ Second, the committee qualified its role as an advisory body responsible for overseeing the uniform implementation of guidelines at the European level. This task had to be reached through the EMBC channels and in collaboration with ESF, by providing policy and technical advice and by organizing common services, such as training courses and a voluntary registry of rDNA research in Europe.

The connection between EMBO’s risk assessment program and the European approach to the US guidelines was emphasized by Weissmann in a letter sent to the NIH Deputy Director for Science DeWitt Stetten just a few days after the first, February 1976 meeting of the EMBO SAC. While sharing the minutes of the meeting, Weissmann noted that EMBO would consider “any further tightening” of the forthcoming NIH guidelines “unwarranted by the limited evidence that is at hand.” Therefore, Weissmann argued, it was a “matter of urgency” to carry out “experiments specifically designed to provide information on which an objective assessment of possible hazards may be made.”^42^ By using scientific international competition as political leverage, EMBO influenced the drafting of the NIH guidelines in the very moment of their final elaboration.

EMBO’s influence on the drafting of the NIH guidelines did not pass unnoticed at the time. In a report on the evolution of the American guidelines in April 1976, science journalist Nicholas Wade, an enthusiastic promoter of the new genetic technologies,^43^ described the political constraint that EMBO could exercise on the drafting of the NIH guidelines as a kind of “veto power,” an issue also taken up and bemoaned on other occasions.^44^

While endorsing and influencing the NIH guidelines, issued on June 23, 1976, and published on July 3 in the *Federal Register*, the EMBO SAC began developing its coordinating action on rDNA regulations at the European and global level. Many members of the EMBO SAC were appointed in their respective national committees and were thus well-positioned to lobby their governments on the subject. Furthermore, they established formal relationships with the ESF ad hoc Committee on rDNA Research (Genetic Manipulation),^45^ the WHO Advisory Committee on Medical Research,^46^ and the ICSU Committee on Genetic Experimentation—COGENE.^47^ In all these contexts the EMBO SAC acted as “a technical advisory committee” for Western Europe. In April 1976 the EMBO SAC received from the Netherlands the first request to advise on a specific local experiment designed by Richard A. Flavell of the University of Amsterdam. Tooze emphasized the importance of this moment in his correspondence with the other members of the committee: “This is the first time that the EMBO Committee has been formally approached for advice by a national committee and I think that the credibility of the EMBO Committee, at least in the Netherlands, will depend very much upon the way we reply to this enquiry.”^48^ The role of the EMBO SAC was significantly challenged in the summer of 1976, when the UK report of the Working Party on the Practice of Genetic Manipulation (the so-called “Williams Report,” from the chairman Robert Williams, director of the Public Health Laboratory Service) was officially published. Compared to the NIH counterpart, the standards of physical containment of the Williams Report were more stringent, while biological containment was less detailed. The Williams Report was more flexible in the approach to rDNA experiments, basically devising a case-by-case procedure entrusted to a national board, the Genetic Manipulation Advisory Group (GMAG), which included not only scientists but also representatives of other grades of laboratory employees and the public. Moreover, the UK code of practice left much responsibility to the laboratory safety committees and gave greater emphasis to the selection, education, and training of all staff working in a containment laboratory.

The presence of two different sets of guidelines, respectively in the US and the UK, not only called for some decision, but also made the lack of European coordination apparent. It was at this point that EMBO tried to become the central *political* body responsible for the regulation of rDNA research in Western Europe, directly competing with the ESF.

Responding to a specific request coming from the ESF and from the EMBC state members, the EMBO SAC produced a detailed comparison between the two systems, deciding in the end to leave each country the option to choose between the NIH and the UK guidelines. In September-October 1976, the ESF ad hoc Committee on rDNA Research (Genetic Manipulation) recommended the adoption in Europe of the Williams Report, while suggesting the creation of a European Committee under the aegis of the ESF, including the national bodies for rDNA research, the EMBO SAC on rDNA, the European Medical Research Councils, and the representatives of agricultural research. A few weeks later, during the presentation of the EMBO SAC report to the EMBC governments, EMBC Secretary General, Arthur Rörsch proposed transforming the EMBO SAC into a proper European Committee—i.e., the peak advisory body for rDNA work in Western Europe. Faced with opposition by the UK, which clearly favored the ESF while considering the EMBO SAC just as “the repository of scientific and technological wisdom in the area concerned,” the EMBC decided to defer a review of the EMBO SAC’s terms of reference. A standing “European Committee” was created under the auspices of the ESF in January 1977. It is interesting to note that this ESF Liaison Committee for Recombinant DNA Research included representatives of EMBO, the NIH, and the Canadian Medical Research Council. In this context, the EMBO SAC acted as technical advisory body for the ESF (Scherwell 1977).

Despite failing to assume a more central political role in coordinating the rDNA research regulation in the Western European context, between 1977 and 1979 the EMBO SAC made a fundamental impact on the revision process of the 1976 NIH guidelines and on the concurrent relaxation of the European regulatory frameworks.

Drawing from the new scientific consensus concerning the safety of cloning in *E. coli* K12 laboratory strains, the EMBO SAC, in its fourth meeting of November 1977, recommended a significant relaxation of the containment measures for rDNA experiments in general, and those involving the use of *E. coli* K12 as the host organism, in particular. The EMBO SAC especially criticized the excessive stringency of the NIH containment for cloning viral DNAs, asking for the establishment of an international ad hoc group of virologists to consider the issue further.

The EMBO SAC’s call for relaxation of the NIH guidelines had a dual effect. First, by December 1977, France’s main research body drafted a set of guidelines whose general containment conditions were very similar to those recommended by EMBO.^49^ Following this approach, both the EMBO SAC and the French Control Commission approved an experiment proposed and performed by Philipson and Pierre Tiollais, involving recombinant fragments of adenovirus DNA with a host-vector system developed at the Pasteur Institute in a P3 (moderate risk) facility. At the time such experiments were only permitted in the United States under the most stringent P4 containment conditions. Significantly enough, the draft of the French guidelines was made available to the NIH before the public hearings held on December 16–17, 1977, to discuss the revision of the NIH guidelines.

Secondly, EMBO’s criticism against the excessive rigidity of the 1976 NIH guidelines was reinforced and communicated through Tooze’s presence at the December 1977 NIH hearings. On this occasion, Tooze reported the EMBO SAC’s position on the stringency of US regulations, proposing once again a joint European-American effort to reconsider the containment levels for the cloning of animal virus DNA in *E. coli*. The European call for regulatory relaxation, along with the risk of the US falling behind in the promising field of rDNA research, reached the American public through the *New York Times*: “A British scientist invited to the meeting said that in Europe studies were already in progress that were still barred here because American guidelines required elaborate laboratory safety facilities that were not yet available” (Schmeck 1977). Unsurprisingly, the NIH accepted EMBO’s suggestion. Just over a month after the NIH hearings, a joint US-EMBO Workshop to Assess Risks for Recombinant DNA Experiments Involving the Genomes of Animal, Plant, and Insect Viruses was held in Ascot, Berkshire, not far from the famous racecourse. Along with Tooze, the workshop was organized by Malcolm Martin and Wallace Rowe, who were working on rDNA risk assessment experiments at the US National Institute of Allergy and Infectious Diseases. Selected virologists coming from the US, the UK, West Germany, Finland, France, Sweden, and Switzerland attended the meeting, while GMAG was not officially invited to participate (Wright 1994, p. 315). After three days of intense discussions, the Ascot Workshop concluded that the cloning of viral DNA posed no greater risk than working with the infectious virus or its nucleic acid, and in most cases presented less risks. Consequently, the participants recommended a significant reduction in containment requirements for experiments with most viruses. Just a few months later, in May, the ESF Liaison Committee endorsed the conclusions of the Ascot Workshop, implicitly seconding what Wright characterized as “the American drive to reduce containment levels for experiments with viral DNA” (Wright 1994, p. 330).

Interestingly, the ESF meeting sparked some tensions that confirmed the strong relationship between EMBO and NIH around rDNA experimentation, as well as their coordinated efforts to relax the US guidelines. In an article in *Nature* dedicated to the meeting, it was incorrectly reported that the NIH was attempting to “mislead” the European counterparts about the forthcoming revision of the guidelines (Walgate 1978, p. 331). In response, Tooze immediately condemned the “stupid and destructive article,” urging the ESF President, Brian Flowers, to clarify the positions of ESF and EMBO to the NIH: “Throughout the work of the Liaison Committee,” Tooze argued, “the NIH has been totally cooperative, frank, and friendly; indeed more cooperative and willing to provide more information than virtually any European GMAG.”^50^ As a result of pressure from EMBO and ESF, *Nature* published a letter of complaint in July, written by ESF Secretary General Friedrich Schneider, reaffirming the “good transatlantic relationships” with the NIH (Schneider 1978). Tooze’s and Schneider’s concerns were soon to dissolve. With a few changes, the Ascot recommendations were incorporated into the revised NIH guidelines, released in December 1978.^51^ In addition, the new guidelines suggested shifting the burden of proof to those advocating special precautions.

In their next meeting in December 1978, the EMBO SAC welcomed the NIH revised guidelines and endorsed them as the new regulatory framework for the Western European context. One year later, at its meeting in February 1980, the EMBO SAC reckoned that the time was ripe for “a radical simplification” of guidelines for rDNA research, providing a clear distinction between “the vast majority of experiments” with insignificant risks and the “very few for which a scientific case for a possibly significant biohazard can still be made.”^52^ To reinforce this position, the committee could now also refer to the results of the “EMBO Risk Testing Experiment” concerning the measurement of the infectivity in tissue cultured mouse cells of rDNA molecules containing polyoma virus DNA. After 2 years of complex implementation, involving protocol approval from the British GMAG and collaboration with the ICRF in London and the Microbial Research Establishment (MRE) in Porton Down, the experiment showed that none of the recombinants tested was as infective as polyoma DNA itself, and the majority were significantly less so (Fried et al. 1979).

From this perspective, the EMBO SAC not only successfully argued for the weakening of the NIH guidelines^53^ but also urged that any discrepancies between US and European regulatory frameworks resulting from NIH decisions would be swiftly removed, as more stringent guidelines in Europe would place molecular biologists “at a disadvantage.”^54^ Accordingly, the committee backed any relaxation of Western European guidelines that aligned with the 1979 NIH guidelines (for instance in France and in Switzerland). Conversely, they advised against adopting the new GMAG probabilistic scheme of risk analysis, devised by Sydney Brenner for the UK,^55^ and opposed any attempts to introduce “new national legislation specifically designed to regulate recombinant DNA research,”^56^ as in the case in West Germany and Denmark.

## Building Containment Facilities and Launching rDNA Research at EMBL (1974–1981)

While the EMBO SAC continued to push for the relaxation of the regulatory guidelines in both the US and Western Europe, a high-risk containment facility was under construction in Heidelberg. How could the construction of a building that could possibly become obsolete within a few years be justified? To address this apparent paradox, this section will explore how concerns about rDNA safety influenced the EMBL project, ultimately reinforcing the justification for EMBL’s establishment and its role as a service laboratory for European scientists.

Timing is crucial here. On July 4, 1974, the international agreement establishing the EMBL in Heidelberg was ratified by a sufficient number of member states, granting the laboratory legal existence. Kendrew, who had pursued the laboratory project with unique energy for many years, was appointed as the first Director-General. The EMBL research policy, defined in 1970 after years of intense negotiations, privileged structural studies and instrumentation (de Chadarevian 2002, p. 332). As the laboratory was on the brink of becoming a reality, the waves of controversy about rDNA in the United States reached the other side of the Atlantic, inevitably challenging the EMBL’s philosophy and design.

On June 7, 1974, the previously mentioned Weissmann-Philipson letter to Kendrew from the Cold Spring Harbor Tumor Viruses Symposium urged the EMBO Council and EMBL Provisional Scientific Advisory Committee (PSAC) to evaluate the establishment of “appropriate special risk laboratories within the framework of EMBO, for instance at the Heidelberg laboratory” to enable rDNA research.^57^ Kendrew’s response, following the EMBL PSAC meeting of June 21, was rather vague, highlighting the need to find “additional budgeting and additional staff” to “erect special and separate facilities in Heidelberg.”^58^ Having negotiated for over 10 years for the agreement of the laboratory now in construction, it is understandable that Kendrew did not show too much enthusiasm to go back to the drawing board.

On July 8 Weissmann expressed frustration over the lack of “positive action” from Kendrew and the EMBL PSAC. Such passivity, Weissman argued, put European molecular biologists at risk of competitive disadvantage compared to their American colleagues: “I think the response by the PSAC has not been very helpful. The scientific community remains without the possibility of carrying out experiments which are of great scientific interest but which are considered out of bounds and have been ‘banned’ by a fairly small group of colleagues.”^59^ Four days later, in another letter to Weissmann, Kendrew provided two reasons for his position: first, facilities for the “DNA grafting experiments” proposed by Weissmann for the EMBL would require “persuading the Governments to put up extra money;” second, they could not be conducted “without an unacceptable distortion of the purposes and research plans of the Laboratory.”^60^ Thus, in addition to budgetary constraints, research priorities in designing the EMBL in Heidelberg were clearly at stake. While EMBL prioritized structural studies, the emergence of the rDNA technology implied growing relevance for molecular genetics and cell biology research. In their interviews with Weiner, respectively in 1975 and 1977, Weissmann and Philipson confirmed the existence of these internal tensions:

Weissmann: Well, Kendrew is, of course, a man who is extremely knowledgeable in physical chemistry, X-ray crystallography, and all things that go into physical work. His specialty is just simply not biology. And he may not have been too well-advised at the time they answered this letter.^61^

Philipson: I approached Kendrew because I had just been elected to serve on the EMBO Council. I then found out that the plans for the EMBO lab were vague and emphasized structural studies. Cell biology was neglected. I approached Kendrew and declared I’d be willing to recruit people and set up a lab. Kendrew, however, wanted to direct the lab himself. He didn’t want to have anyone from the outside coming in and trying to help him to establish the EMBO lab. We saw another opportunity to convince him at the Tumor Virus meeting in Cold Spring Harbor in 1974.^62^

Weissmann’s and Philipson’s action, along with informal conversations with Brenner and Jerne, and solicitations from several EMBC delegates (Borsellino for Italy, Kjeldgaard for Denmark, Peter Reichard for Sweden, Zachau for West Germany),^63^ persuaded Kendrew to change his mind on the subject and begin collaborating with Paul Berg in the organization of the Asilomar Conference, while considering a revision of the laboratory plan.

At its first meeting in December 1974, the EMBL Council discussed the issue of “gene transplantation (genetic engineering).”^64^ On this occasion, the delegates of Switzerland, Netherlands, France, and Israel encouraged EMBL to become the European reference point for research on rDNA in high security facilities, either by establishing an outstation within the EMBL program or by building new containment rooms in Heidelberg. Faced with these national requests, Kendrew’s reaction was positive yet cautious: “The main problem,” he constantly repeated to the delegates, “was the constraint in space and budget.”^65^ A few days later, at the meeting on January 10, the EMBO Council decided that the EMBL Scientific Advisory Committee (SAC) would be urged to consider, in the light of the forthcoming Asilomar Conference’s recommendations, “what changes in the design of the EMBL building should be made, to enable hazardous work on hybrid DNA molecules to be carried out.” Governments—the EMBO Council added—“should be asked to provide an additional budget to make this possible.”^66^

The suggestion to establish a centralized containment facility for rDNA research in Heidelberg gained momentum following the Asilomar Conference. As previously noted, on July 3, 1975 the EMBO delegation to the Asilomar Conference presented its report to the EMBC member States. By emphasizing the fortuitous “coincidence of the development of Recombinant DNA technology with the establishment of the European Molecular Biology Laboratory,” the report proposed setting up a high-risk containment laboratory in Heidelberg to promote European collaboration in this research area. It further argued that the permanent staff of EMBL should include “a strong group of scientists” actively working in rDNA research, and that these changes to the EMBL project were “a matter of urgency” if Europe wanted to keep up with the United States.^67^ On that same day, the EMBL SAC unanimously recommended that facilities for medium- and high-risk experiments with rDNA be built in Heidelberg and that EMBL embark on research in this field as soon as possible. Two reasons justified this position: first, the Laboratory should provide a “service facility” for national laboratories in Europe, thus becoming “the proper place to train new workers in this field;” second, if the Laboratory wanted to be at the forefront of molecular biology, a project in “genetic engineering” should be started as soon as possible.^68^

Following this recommendation, Kendrew proposed to national delegates at the second EMBL Council meeting, also held in Heidelberg on 3–4 July 1975, to erect a special hut of about 400 square meters with medium- and high-risk containment facilities. This could not be incorporated in the design of the EMBL permanent building without delaying the construction of the laboratory by at least a year. The estimated cost for a separate “genetic engineering” hut was around 1.7 million German Marks (DM), approximately equivalent to US$690,000 at the time.^69^ During the following discussion, the scientific adviser of the UK delegation to the EMBL Council, structural biologist David Chilton Phillips, stated that the MRE in Porton Down was prepared “to make its facilities freely available to the workers from European countries to do experiments in the field of genetic engineering,” stressing the fact that the staff there was already “well-trained in handling dangerous micro-organisms.”^70^ However, all other delegations supported the establishment of a containment hut in Heidelberg. The general sentiment was epitomized by the intervention of the Israeli adviser Yaakov Saphir, deputy director of the National Council for Research and Development in Jerusalem, who strongly favored the Heidelberg solution, “both because it was a field which would lead to important progress in molecular biology and because an international laboratory would be in a strong position to establish standards to be followed by the national laboratories.”^71^ Furthermore, in his interview with Weiner, Kendrew mentioned that most EMBL delegates did not favor the idea of launching a research program in rDNA behind the gates of a UK defense establishment like Porton Down.^72^

The final decision of the EMBL Council included three points: that a genetic engineering program should be started at EMBL as soon as possible, “preferably before the establishment of the final laboratory;” that this program should be carried out at EMBL; and that the Director-General of the Laboratory should investigate the financial aspects of the project, while consulting the EMBO SAC and the EMBL SAC. The delegates agreed on this summary, except for the UK that contested the lack of information about the scientific and economic impact of the Heidelberg solution. The British delegation—it was reaffirmed—wished “to be convinced that the project suggested could not easily be carried out in existing facilities like those at Porton, Pirbright or Tübingen.”^73^

In November 1975, Kendrew submitted the proposal for “an EMBL facility for genetic engineering” to the EMBL Council.^74^ In the introduction, the proposal emphasized the potential applications of the rDNA technology in medicine, agriculture, and industry, citing examples such as the large-scale production of medically important substances (insulin, antibiotics, growth hormone), the improvement of nitrogen fixation in crops, and the “correction of hereditary defects in human beings.”^75^ The lack of containment facilities in many European countries, along with the increasing necessity to train research workers and technicians in the use of dangerous pathogenic organisms, justified the decision to establish a centralized containment facility in Heidelberg.

Kendrew’s project comprised two elements. The first envisaged the implementation of a strong in-house research program in rDNA. This was conceived not only as part of the cell biology program of the Laboratory but as a fundamental precondition to provide “adequate services” to external visiting groups. These services would include maintaining safety standards, instructing visiting groups in safety precautions, preparing restriction enzymes, maintaining a stock of appropriate strands of bacteria and animal cell lines, providing and designing suitable equipment, and offering general scientific advice and collaboration.^76^

The second part of the proposal was dedicated to the description of the containment facility to be built in Heidelberg. This was conceived as a special hut of 700 square meters, with 200 square meters of medium- and high-risk laboratories. Cost and time scales were based on figures supplied by consultants involved in operating containment facilities in Europe, namely the MRE in Porton Down, the *Landesimpfanstalt* in Munich, and the *Bundesforschungsanstalt für Viruskrankheiten der Tiere* (BFAV) in Tübingen. Building constructions and laboratory furniture amounted to 5 million DM approximately (around US$ 2 million), while recurrent costs amounted to 2.3 million DM (around US$ 1 million).

Crucially, the EMBL Finance Committee assured the Council that the costs of the “Genetic Engineering Program” could be covered without adding new resources to the Indicative Scheme approved by governments.^77^ With no additional costs expected, the EMBL Council approved the project, simply authorizing the budgetary arrangements needed for its financing.^78^

To discuss the design of the containment facility and define an EMBL research program on rDNA, a working group was established, including some external advisers, notably Mark Darlow, a medical and microbiological safety officer at Porton Down.^79^

Work on the containment facility progressed swiftly. The hut was completed in November 1977, even before the official inauguration of EMBL.^80^ To make it fully operational and to reassure the increasingly worried local press,^81^ at the end of 1977 EMBL hired Hotse Bartlema, from the Medical Biological Laboratory in Rijswijk, as Microbiological Safety (or Biosafety) Officer. After extensive training during a series of visits to facilities in Europe and United,^82^ Bartlema became responsible for testing the EMBL containment facilities and for training personnel using the facility through a set of “house rules” and the organization of experimental courses in medical microbiology.^83^ Furthermore, in July 1978 two specific Committees were set up: a Priorities Committee, international in character, charged with recommending priorities for the acceptance of projects proposed by visiting researchers, and the appropriate levels of physical and biological containment; and a Safety Committee, charged with monitoring the safety procedures in collaboration with the Central Commission for Biological Safety (*Zentrale Kommission für Biologische Sicherheit*, ZKBS).^84^ Importantly, because the laboratory was located in Germany, it had to comply with German safety rules in the matter. Although German guidelines did not require the existence of local safety committees, Kendrew and the EMBL SAC thought that “owing to the special situation of EMBL it was important that such a Committee exists.”^85^ With the agreement of the ZKBS, experimental work in P3 containment in Heidelberg began at the start of 1979.^86^ The downgrading of German guidelines made P4 capacity scarcely significant, as not a single request for work under P4 conditions had reached the EMBL by November 1980.^87^ Meanwhile, many EMBL member states had constructed their own P3 facilities. Despite this, the EMBL Council decided to retain some P4 capacity in Heidelberg for European countries which did not (or did not want to) have such facilities.^88^

Along with the building of the containment facility, the rDNA scientific staff grew rapidly. In the summer 1977, three research groups working on rDNA joined the EMBL Cell Biology Division, followed shortly by a fourth group.^89^ By the end of 1978, Ken Murray was appointed as EMBL senior scientist and head of the containment facility.^90^ His wife Noreen, also a distinguished researcher in rDNA, joined EMBL in February 1980,^91^ marking the completion of the rDNA staff recruitment. At this point, Kendrew’s opinion was enthusiastic, as reported in the EMBL Council meeting minutes: “One always had the impression that there were more people, post-docs and visitors, working in the containment facility than there had been the week before, and in his [Kendrew’s] view a crowded laboratory was a very good sign.”^92^ According to Kendrew, the development of an intense research program on rDNA showed to EMBL member states that the investment in the hut had not meant wasting “a lot of money” on a facility that would attract little use.^93^ On the contrary, as the June 1981 Director General’s report emphasized, “it was probably the most crowded part of the Laboratory.”^94^

To sum up, it is worth noting that echoes of the post-Asilomar biosafety controversy resonated at the EMBL inauguration ceremony, on May 5, 1978. On this occasion, the President of the Federal Republic of Germany, Walter Scheel, hailed the establishment of the laboratory on the hills of Heidelberg as a “new chapter in the history of biological research,” the revolutionary opportunities of which demanded greater responsibilities:

The opportunities and hazards of this research are well known. We are aware that the results of this research can have far-reaching and possibly revolutionary implications for human life. By shelving specific research work on new combinations of genes, scientists have emphatically drawn attention to these consequences, whilst at the same time acknowledging their responsibility for the effects of biological research. We would welcome it if this approach could serve as a model for future research planning.^95^

This was not just presidential rhetoric. As we have seen, the Asilomar Conference and the subsequent discussion over research guidelines had a major impact on the early development of EMBL. Firstly, it reinforced the concept of the European laboratory as a provider of services for the member states: from the outset, the containment hut was in fact conceived as a “service operation” that would benefit the development of molecular biology in Europe while closing the gap with the United States.^96^ Secondly, it triggered a significant shift in EMBL research policy, with rDNA research assuming a central role in the development of the Cell Biology Division of the laboratory.

## The Asilomar Legacy and the “Future of EMBO” (1975–2004)

Beyond its actual impact on European biotechnology policies, the experience of the EMBO SAC was itself offered as a model of what EMBO defined as its advisory function. The lesson of Asilomar was thus reshaped to bolster EMBO’s role as an expert body for European science policy.

To investigate this long-lasting legacy of the rDNA debate, this section will focus on the programmatic documents that EMBO elaborated every eight years, between 1978 and 2002, that obtained renewed funding from the EMBC. We have considered EMBO’s involvement in the establishment of the European Research Council (ERC) between 2000 and 2006 as a coherent endpoint for our analysis.

The titles of the four documents considered here were very similar, and all involved a certain degree of speculation about the “future of EMBO:” *The Future of the General Programme Beyond 1980* (1978); *Proposal Concerning a Prolongation of the EMBC Agreement Beyond April 1988* (1986)*; The Future of EMBO* (1994-95); and *Planning Document on the Future of the EMBC/EMBO; Building on a Record of Achievement* (2002)*.* These documents were periodical attempts to envision EMBO’s purpose by responding to a number of recurring questions over three decades: What was molecular biology? What was EMBO’s role? What warranted the special consideration of one discipline? How could EMBO contribute to the development of the life sciences in Europe?

The link between the “Asilomar legacy” and the discussion of the “future of EMBO” can be discerned in the March 1976 interview of Tooze with Weiner. On that occasion, Tooze reflected on the Asilomar Conference with the benefit of hindsight, viewing it as an important scientific and educational event that had played a key role in stimulating rDNA research in Europe. However, he was critical of Asilomar’s attempt to establish guidelines: “The bulk of the people there were perhaps ignorant both of the potentialities of the science itself—I mean in any detailed way—and were also more or less totally ignorant, as is almost everybody else, of the pathogenicity of *E. coli*. So that perhaps it did the best it could.”^97^ More significantly, in Tooze’s view, the Asilomar Conference highlighted the tension between the short-term decision to regulate the safety of rDNA technologies and the long-term ethical and political implications of “synthetic genetics” more broadly.^98^ This, in turn, raised another dilemma: should scientists with technical expertise actually be “the ones who are in any way involved in drawing up policy decisions?” For Tooze, this question remained “open to debate.”^99^ Not surprisingly then, when asked about EMBO’s role in the broader discussion concerning the social and political issues related to rDNA technology, he responded somewhat dismissively: “I would like to think there was a role for EMBO in these future developments and future questions of a sort of general and broad and political and social nature. I don’t actually think there is in practical terms. I think EMBO may be very lucky if it survives as it now stands beyond 1980, just as a funding agency for molecular biology for research.”^100^

A couple of months before this interview, in anticipation of the debates over the prolongation of the EMBC Agreement in 1978-80, Tooze had established a small “ad hoc Committee on the Future of EMBO.^101^ In 1977, the item “Future of EMBO” was included in the agenda of the twenty-sixth EMBO Council meeting. In preparation of the meeting, Tooze drafted a document, entitled *EMBC Retrospect and Prospect*. The situation,Tooze wrote, was “critical:” the flourishing economies of the 1960s were over, and possible cuts of the EMBC budget should be expected. For these reasons, the members of the EMBO Council had to take direct action at the national level, assuming the same diplomatic role that had proved so successful 10 years earlier for the establishment of the EMBC.^102^ Introducing the discussion on this topic at the Council meeting, the EMBC Secretary General, Arthur Rörsch, made no secret of his concerns.^103^ The future of EMBO beyond 1980 was “difficult to predict:” contributions to the budgets of EMBC and EMBL were to be found from national research councils “at a time when these budgets were being reduced,” he reportedly said.^104^ In order to face this difficult situation, Rörsch proposed to the Council a number of possible initiatives, drawing particular attention to the new public role that EMBO had assumed with regard to the rDNA debate:

In order to ensure the future of the General Programme of the Conference, Prof. Rörsch felt it would be essential to prepare an indicative scheme with new scientific dimensions; to perhaps give more weight to the EMBO Standing Advisory Committee on Recombinant DNA, to encourage new countries, perhaps East European countries, to join the EMBC, and finally to establish further collaboration with the European Science Foundation.^105^

To draw public attention to EMBO’s activities, the Council decided to prepare “an outright public relations campaign.”^106^ Additionally, an “Ad hoc Committee on the Future of EMBO” was charged to draft a programmatic document to be discussed at a special EMBO Council meeting of September 1977. For this occasion, the EMBO Council members provided a detailed description of the “new achievements of molecular biology.” These contributions were instrumental for reiterating the case of a “special treatment for molecular biology” at the European level and for justifying the need to maintain the EMBO/EMBC channels of funding.^107^ Discussing future EMBO activities, the Council proposed forming a series of peer review panels “to assist national authorities in the assessment of national research proposals” across Europe.^108^ The definition of a new role for EMBO as a “peer review group” was explicitly inspired by the experience of the EMBO SAC: “The EMBO Standing Advisory Committee on Recombinant DNA provided an example on how EMBO’s expertise had been utilized by national agencies, in their efforts to draft and to harmonize guidelines for in vitro recombinant DNA research.^109^ In 1978, the EMBC approved the document on the “future of EMBO” and agreed on the prolongation of the Agreement. With regard to EMBO peer-review panels, the Conference endorsed their implementation but on the budget of the requesting countries.^110^ Tooze commented that “[a]ll in all it was a fairly good result, considering the economic climate.”^111^

Thus, between 1975 and 1978 the EMBO SAC’s usefulness was held up as an example that was instrumental in legitimizing EMBO’s request for the first eight-year prolongation of funding, and inspired a new advisory function to be implemented in the future. Not surprisingly, then, in January 1980, the EMBO Council decided that the Committee would be “‘moth-balled’ rather than dissolved, because one could not entirely predict future developments.”^112^ During the 1980s, the emergence of the European Community as a major actor in genetic engineering regulation put the EMBO/EMBC advisory function to test. The new proposal that EMBO submitted to the seventeenth EMBC ordinary session in 1986, asking for another eight-year prolongation of the Agreement, celebrated a new “era of extraordinary advance” in molecular biology, made possible by gene cloning, DNA sequencing, and production of monoclonal antibodies.^113^ In the document, EMBO’s advisory function in providing “international peer review” was referred to in very broad terms.^114^ The debate over GMOs and their release in the environment, in Europe and particularly in Germany, would re-activate EMBO’s ambition to work as an “expert body.”

In the mid-1980s, the regulatory framework in Europe regarding biotechnologies appeared very fragmented. The only tangible European piece of regulation produced was the Council recommendation (not a Directive) adopted in June 1982, asking for national systems of notification (not authorization) of rDNA work. In 1986, a Group of National Experts (GNE) of the Organization for Economic Co-operation and Development (OECD) approved a set of safety guidelines (popularly known as the Blue Book), according to which there was “no scientific basis for specific legislation to regulate the use of recombinant DNA organisms.”^115^ At the level of the national states, the situation varied significantly, ranging from systems of monitored self-regulation in the UK and France to the very strict, process-driven Gene Technology Act, approved in Denmark in 1986.

During the second half of the 1980s, two critical developments contributed to reshape this framework. First, West Germany soon became the arena of a heated political and public debate about genetic engineering, leading to the approval of a rather stringent Gene Technology Law (*Gentechnikgesetz*), in May 1990. Secondly, during the second half of the 1980s the European Community (EC) entered the stage by adopting two directives (90/219 and 90/220), one concerning the “contained use” of genetically modified micro-organisms, the other one the “deliberate release” of such organisms. Both focused on the techniques of genetic modification rather than on the properties of the organisms generated or, in legislative parlance, on the process rather than the product.

In this context, the newly appointed EMBC Secretary General Werner Franke, a professor of cell and molecular biology at the German Cancer Research Center in Heidelberg, sought to get EMBO and EMBC directly involved into the political debate on regulation. Franke was particularly concerned about the German political context and its impact on EMBL and EMBO. In July 1988, at the first part of the 19th EMBC ordinary session, he invited all delegations to report on their respective national situations with regard to rDNA research. “It would be very unfortunate,” Franke declared, “if different Member States of the Conference adopted different legislation or other administrative procedures to handle this research.”^116^ The reactions of the delegates only confirmed Franke’s concerns. The general situation in Europe was rather differentiated. Many EMBC delegates pointed out that the question of regulation of rDNA research was already being discussed by the OECD and the EC.

Facing this complex situation, how should EMBC proceed? First, the Conference declared that it had no authority to discuss rDNA legislation since it was composed of government representatives who were not in a position to comment—adversely or otherwise—on national legislation of the member states. Second, it was argued that drawing the attention of member states’ governments to the discussions on rDNA research that were taking place in the OECD and the EC would be counterproductive: such a step might be construed as an expression in favor of a specific legislation or regulation. In the end, the EMBC endorsed the solution suggested by the new EMBL director, Lennart Philipson: EMBO had a Standing Advisory Committee on Recombinant DNA—the EMBO SAC—and the Conference could refer to this Committee (or to the EMBO Council) for a qualified opinion.^117^

A few months later, in October 1988, at the fortieth EMBO Council meeting, Franke reported on the discussion at the EMBC session. To respond to the governments’ request, the EMBO Council issued an official statement addressing the EMBC delegations and calling for the harmonization of European rDNA legislation on the basis of a product-oriented framework.^118^

Franke presented that EMBO statement at the next EMBC ordinary session, in December 1988. The EMBC welcomed the EMBO statement, but the dominant mood among the delegations was one of discouragement.^119^ There was not only concern about the impact of the German legislation on EMBL but also widespread pessimism about the possibility to affect political decision-making through scientific argumentation. As Franke noted: “The problem within the scientific community was that it acted too late and that its voice carried too little weight with legislators.”^120^ The scientific representative of the German delegation, virologist Walter Doerfler of the University of Cologne, shared this same opinion: “Advice from scientists against legislation was ignored.”^121^

On May 16, 1989, the EMBO statement, with a covering letter, was transmitted to members of the European Parliament by Philipson and Max Birnstiel, Chairman of the EMBO Council. The European Parliament was invited to seek the help of the EMBO SAC in the evaluation of legislation concerning “contained use” and “environmental release” of genetically modified organisms.

EMBO/EMBC attempts to provide technical advice to the European Parliament and influence the discussion concerning the approval of the EC Directives turned out to be unsuccessful. On November 23, 1989, Franke resigned from the role of EMBC Secretary General on a twofold motivation. First, EMBO’s statement on rDNA had been ignored by the Ministry of Research and Technology of the Federal Republic of Germany. The document had not even been forwarded to the competent parliamentary committees. As a result—and this was Franke’s second reason for resigning—the German government as well as major political parties, such as the Social Democratic Party and the Green Party, continued to treat molecular biologists “categorically as irresponsible and immoral scientists” whose research had to be “systematically controlled and approved of by non-scientists, notably politicians, theologists and jurists.” According to Franke, this kind of oppressive state control was totally disrespectful of molecular biologists’ autonomy. Historical precedents could only be found in the Galileo affair, the ban against Charles Darwin in the US, or the “Lysenko controversy” in the Soviet Union.^122^

One week later, Franke’s letter of resignation was presented and discussed at the EMBC twentieth ordinary session. On this occasion, tensions eased: the German delegation apologized to Franke, declaring that an office mistake had resulted in the failure to distribute the EMBO Council’s statement to relevant committees of the German Parliament. In turn, Franke recognized the misunderstanding, and withdrew his resignation.^123^ Albeit quickly settled, the situation offered a chance to discuss once again EMBO’s advisory function. At the end of the discussion, Philipson re-affirmed the usual strategy: EMBO had a Standing Advisory Committee on rDNA, and the European Parliament had been invited “to seek its help in the evaluation of such legislation.”^124^

Adopted in April 1990, the two EC Directives 90/219 and 90/220 advocated a process-based, horizontal, and precautionary approach to regulation: the exact opposite of what the EMBO Council had suggested. Despite having lost the political battle regarding legislation, regulation and control of genetic engineering and GMO products, EMBO did not give up its aspiration to act as an expert body in the European arena. On the contrary, in the 1990s EMBO’s approach to scientific expertise was reframed in order to emphasize the key role the organization could play in the implementation of a European research policy in molecular biology.

In this context, in September 1995, the EMBO Council decided that the “Recombinant DNA Committee” (the EMBO SAC) would be “disbanded;” the Committee “had fulfilled a very important role at one stage but had been inactive for a number of years.”^125^ However, the role played by the EMBO SAC continued to be evoked as a guiding model for discussions on EMBO’s policy functions. In 1991, in a commentary published in *Nature*, Philipson proposed that EMBO provide a service of international peer-review at the European level to mitigate the “increasing polarization and competition between the different European organizations.” In this article, Philipson recalled EMBO’s role in the rDNA controversy as a crucial precedent, highlighting it as “an example to national legislators of how to separate science from fiction” (Philipson 1991). This suggestion resurfaced in the lead-up to the renewal of the EMBC Agreement in 1996, outlined in the programmatic document *EMBO—The Future*, prepared by Frank Gannon, newly appointed EMBO executive secretary.^126^

In the introduction to this report, Gannon stressed the urgent need for change.^127^ As the fruits of research in molecular biology were rapidly moving from the laboratory through industry and into society, and fuelling increasing distrust and hostility towards “novel therapeutic approaches” or “targeted modification of genes in plants and animals,” EMBO was more than ever called upon to play a crucial role in countering “unfair criticism” and providing an objective evaluation of risks and benefits. Gannon’s reference explicitly turned to the 1970s and the experience of the EMBO SAC on rDNA:

A new role for EMBO could be that of *preparing fact files on these new developments*, of *identifying the real risks*, of indicating the steps which have been taken or should be taken to overcome them, of pointing out the limits of the methodologies and of indicating the benefits which can arise from them. *This would be in keeping with the role it played in the 1970s through its Recombinant DNA Committee*.^128^

In addition to this political and public role in support of genetic engineering in Europe, EMBO had to expand its function of serving as an international peer-review group, broadly announcing its availability to act as a scientific base for the provision of reviews and advisory councils.^129^

The discussion of Gannon’s document during the EMBO Council meeting of September 1995 led to the creation of a new Committee on Public Affairs and Awareness (renamed the Science and Society Committee in 1997). The committee organized annual meetings in different EMBC member states,^130^ and it produced policy papers on matters of relevance to molecular biology.^131^ The peer-review activity gained momentum in the mid-1990s. A Peer-Review Committee was established in 1993, together with a specific set of procedures.^132^ It conducted surveys on the state of molecular biology in Austria (1994–1995), Finland (1996–1997), and Hungary (1997–1998), while providing peer-review in the selection of research grants (Portugal, 1997) and post-doctoral fellowships (Spain, 1997),^133^ as well as in the evaluation of national biotechnological programmes (Genopole, France, 2002).^134^

At the dawn of the new millennium, the debate over the establishment of the European Research Council (ERC) came to reactivate once again the memory of the EMBO SAC. In October 2002, at a conference in Copenhagen significantly titled *Do We Need a European Research Council?*, Frank Gannon suggested that EMBO and EMBC could provide “a greater service to the European Community” as a “prototype” for the ERC.^135^ At the thirty-third EMBC ordinary session, in July–November 2002, a dense programmatic report, prepared by Gannon and the EMBO Council, and entitled *Planning Document on the Future of the EMBC/EMBO: Building on a Record of Achievement*, framed the connection between EMBC and ERC by taking a retrospective look: “The establishment in 1970 of the EMBC de facto established a specialised European Research Council that has not fully fulfilled its potential to date.”^136^ The aim of the *Planning Document* was not only to justify the prolongation of the EMBC Agreement from 2004 to 2013 but also to promote the EMBO/EMBC as a “model” or a “pilot scale” for the ERC.^137^ To reach this ambitious goal, the EMBC had to complete the work initiated by its founders in 1970 and provide funding for research grants, in addition to fellowships, courses, workshops, and lectures.

As potential “new developments” for the future, two possible EMBO/EMBC “combined actions” were mentioned. First, EMBO should act as an “advisory board” for EMBC. According to the *Planning Document*, recognizing EMBO as an “academy,” analogous to the US National Academy of Sciences would benefit both the EMBC governments and the EMBO members: the EMBC would find in EMBO an expert body for decision making in topics of a complex nature; the EMBO members would find “a better channel of communication” for their views as well as a public arena where their social responsibilities could be heightened.^138^ Secondly, EMBO could operate as a “reviewing body,” providing quality assessment for European research institutes willing to improve their performances.^139^

The EMBC delegations and the EMBC Strategic Working Party approved the *Planning Document*, recommending adopting it “as a manifesto for the next actions of the EMBC/EMBO and as the start-up manifesto for a component of a putative European Research Council.”^140^ The transformation of EMBO into an “academy,” that is a sort of collective scientific advisor for EMBC delegations, was considered “not necessarily desirable,” not least because in the European context the term “academy” had “a cultural interpretation that varied from country to country.”^141^ As an alternative, the Strategic Working Party prepared a resolution which would mandate the EMBC to request an “input on science policy” from EMBO, “on an annual basis.” This proposal was motivated by explicitly recalling the past experience of EMBO’s role in the 1970s rDNA debate.^142^

In June 2004, at the thirty-fifth EMBC ordinary session, the resolution was withdrawn as it was considered “an excessive strong device to use to achieve a goal that could be achieved otherwise.” However, the general sense of the resolution—“that EMBO would act as an adviser to the EMBC”—was retained through the lighter mechanism on an “annual input” at the EMBC meetings.^143^ To implement this decision, the EMBC Strategic Working Party suggested that EMBO would arrange “a system of forward look (strategic planning documents)” to the benefit of the governments of all member states.^144^

After almost thirty years, the lesson of Asilomar was thus incorporated in the relationship between EMBO and EMBC as a formal channel of science policy discussion and strategic planning for the future.

## Conclusion

In 1981, James D. Watson, director of the Cold Spring Harbor Laboratory (CSHL), and Tooze published *The DNA Story. A Documentary History of Gene Cloning*.^145^ Initially titled *Recombinant DNA Scrapbook*, Watson and Tooze’s book was conceived as an anthology “of interest to the public, to students of sociology and the history of science, and to molecular biologists.”^146^ Introduced by George Kelvin’s full-color illustrations, which visualized the rise of biotechnology from the double helix structure to the commercial promises of recombinant DNA, the book was described by the authors as “a black and white drama in two acts, beginning with calls for a worldwide moratorium on recombinant DNA research and culminating almost eight years later in a worldwide boom industry based on DNA” (Watson and Tooze 1981, p. XI). The “black-and-white” referred to the documentary aspect of the book, a 600-page collection of primary sources, including official reports, private correspondence, photographs, and cartoons. While claiming to present the history of the “recombinant DNA controversy” in “as impartial a way as possible” (Watson and Tooze 1981, p. XI), the book clearly reflected the authors’ idiosyncratic views. Since 1975, Watson had conducted a cultural and political campaign “in defense of DNA” (Watson, 1977) and against any legislative regulation of rDNA research, regretting his early support of the so-called moratorium in 1973. Similarly, Tooze endorsed Watson’s criticism (Tooze 1978) and worked for the relaxation of the guidelines in the US and in the international context through his multipositional role within EMBO, ESF, and COGENE. Both considered the Asilomar Conference as a good-faith error, framing their historical reconstruction as a paradigmatic struggle between the “Reason” of the scientific community and the “Hysteria” of “the populace;” between the “Molecular Biology Establishment” and “some fringe groups,” such as Science for the People or the environmental organizations; and, ultimately, between Science and Politics (Watson and Tooze 1981, pp. IX–X). Not surprisingly, then, the short “Epilogue” of *The DNA Story* sounded like a sigh of relief after eight years of battle against “unnecessary restrictions and censorship:” “Politics and politicking preoccupied the first years of the recombinant DNA story, but that phase, fortunately, is fast becoming history. This book is our epitaph to that extraordinary episode in the story of modern biology” (Watson and Tooze 1981, p. 584). Never was a prediction more unfortunate, not only because the “politicking” continued well beyond 1981, but also because historians do not typically write epitaphs for supposed graves. From this perspective, *The DNA Story* serves more as a source book for historians of science than as a historical account, as Watson and Tooze’s positions crystallize a crucial turning point in the evolving relationship between the US and Europe. While the NIH and EMBO collaborated in the 1970s to relax rDNA guidelines, the rise of the European Community as a key player in regulating genetic engineering during the 1980s increasingly led to regulatory conflicts between the US and Europe in biotechnology (Gottweis 2005, p. 337).

This article has sought to reopen the historiographical discourse on Asilomar, by focusing on the still-neglected history of what Watson and Tooze, himself a strong operator in the European scene, defined in their book as “the European side shows” of the controversy (Watson and Tooze 1981, pp. 305–309). From our analytical perspective—limited to EMBO, EMBC, and EMBL—the European response had a tangible impact on both the internationalization of the Asilomar Conference and on the history of the NIH guidelines. From 1977 to 1979, EMBO crucially supported the NIH efforts to reduce its controls and prevent the introduction of new regulatory legislation for rDNA research in the United States. At the same time, the Asilomar Conference had a decisive impact on the European science policy scene. Two aspects were particularly salient.

First, Asilomar provided new and vital legitimization for EMBO and EMBL. It proved that the strategy that had prompted the birth of EMBO ten years earlier was fundamentally effective. The rise of rDNA research produced once again the emergence of a perceived gap that required closing. Europe, Tooze declared in March 1976, had “missed the boat to a considerable extent” but “the American boat” wasn’t so far at sea to exclude the “chance of keeping in the race with it.”^147^ Through two complementary actions—the shaping and subsequent dismantling of guidelines in the US and Western Europe, on the one side, and the establishment of a high-risk containment facility in Heidelberg, on the other—EMBO and EMBL provided the transnational framework that Western Europe needed to keep pace with the United States.

Second, along with confirming EMBO’s role in strengthening molecular biology in Western Europe, Asilomar offered EMBO the opportunity to experiment with a new function: acting as a scientific advisory body for Western European governments. The novelty of this emerging role needs some qualification. From its inception, EMBO’s founders had envisioned their association as an advisory board for molecular biology on a European scale. As we have shown, EMBO’s involvement in the Asilomar Conference provided significant momentum for turning this aspiration into reality. One could say that this new science policy dimension came packaged with the recombinant DNA technologies. As the practices of molecular biology evolved, so did the functions of EMBO. Once research in molecular biology became subject to government regulations, scientists had to engage at that level. The aim of promoting molecular biology research in Europe remained, but whereas this had previously meant funding fellowships and establishing a European laboratory, with Asilomar the focus shifted to influencing science policy measures.

This was likely the most enduring impact of Asilomar on EMBO. As this article demonstrates, the EMBO SAC had a longer lifespan than is usually assumed. Although it was “mothballed” in 1980, it remained active until 1996. More significantly, over almost three decades, between 1978 and 2004, it was repurposed as an example and a model to support EMBO’s ambition to become an advisory group for European science policy in molecular biology.

## Data Availability

No datasets were generated or analysed during the current study.

## Footnotes

1 Krimsky (1982) devoted a brief chapter to the Asilomar Conference as an “international affair,” but his analysis remained predominantly US-centered.

2 The international conference *Engineering Life: Regulating Science, Risks, and Society in Europe* (Paris, Rice University-Global Paris Center, 14–16 June 2023), organized by Luis Campos (Rice University), Francesco Cassata (University of Genoa and Centro Linceo “Beniamino Segre”) and Christina Brandt (University of Jena), focused on the European dimension of the Asilomar legacy.

3 In April 1970, when the EMBC Agreement entered into force, the Conference included twelve Western European states (Austria, Denmark, Federal Republic of Germany, France, Greece, Italy, Netherlands, Norway, Spain, Sweden, Switzerland, and the UK), Israel, and the *Fonds National pour la Recherche Scientifique* (FNRS) for Belgium. National delegations included science administrators and leading molecular biologists (often EMBO members). Israel’s scientific, economic, and political involvement in the birth of EMBO motivated its inclusion in a Western European intergovernmental structure. On this see de Chadarevian (2002, p. 327) and Cassata (2024, pp. 37–38).

4 Interview with Weissman by Charles Weiner, May 29, 1975, p. 34, in MIT Oral History Program on Recombinant DNA (hereafter MIT-OHP), Box 14, folder 167. In 1975, shortly after the Asilomar Conference, an oral history project was initiated to ensure the preservation of unique source materials essential for understanding the history of the controversy. The project was directed by Weiner, Professor of History of Science and Technology at MIT. The Recombinant DNA History Collection is the product of that project and is housed in the Department of Distinctive Collections of MIT. The collection consists of interview transcripts, written material, and audio and video tapes of events.

5 Interview with Tooze by Weiner, March 26, 1976, p. 87, in MIT-OHP, Box 14, folder 162.

6 On the history of European science policy, see in particular Krige (2003) and Mitzner (2020).

7 Interview with Berg by Weiner, May 17, 1975, in MIT-OHP, Box 1, folder 8.

8 Interview with Zachau by Weiner, August 29, 1977, p. 1, in MIT-OHP, Box 15, folder 174.

9 During a sabbatical from 1971 to 1972 in Ernest Winocour's laboratory at the Weizmann Institute of Science in Rehovot, Singer initiated a new line of research on the DNA of SV 40. In 1975, she joined the National Cancer Institute (NCI) as the chief of the Nucleic Acid Enzymology Section in the Laboratory of Biochemistry.

10 Dieter Söll earned undergraduate and PhD degrees from *Technische Hochschule* in Stuttgart. He did postdoctoral work with Har Gobind Khorana at the University of Wisconsin’s Institute for Enzyme Research and served as an assistant professor there in 1965–1967. In Khorana’s laboratory, Söll used transfer RNA to determine the codon recognition pattern. In 1967, he joined the Yale faculty as an associate professor in molecular biophysics and biochemistry.

11 On June 21, 1973, Singer wrote all the Gordon Conference participants to confirm their agreement on sending the letter to the NAS and IOM and on its potential publication in *Science* magazine: “Because we are doing these experiments, and because we recognize the potential difficulties, we have a responsibility to concern ourselves with the safety of our coworkers and laboratory personnel as well as with the safety of the public” (see https://web.archive.org/web/20170203084021/https://profiles.nlm.nih.gov/ps/access/CDBBCF.pdf, accessed 12 December 2024).

12 The Committee on Recombinant DNA Molecules also included Ronald Davis, David Hogness, Daniel Nathans, Richard Roblin, James Watson, Sherman Weissman, and Norton Zinder.

13 On July 12, 1974, Arber wrote to the EMBO Council Chairman, Niels Jerne, referring to the Ghent workshop and calling for EMBO’s collaboration with the US NAS in the regulation of rDNA research: “At the recent EMBO workshop on restriction enzymes in Ghent, Dr. Daniel Nathans reported on discussions held in this context by a committee of the US National Academy of Sciences […]. Thinking that the European scientific community will also be concerned with these questions, I would like to propose to the EMBO council to enter in contact with this American group and to investigate whether it would be appropriate for EMBO to collaborate with the US National Academy of Sciences for solutions to the questions raised.” See Arber to Jerne, July 12, 1974, in EMBO Archive, Heidelberg, uncataloged (hereafter, EMBO Archive), box “Correspondence Kendrew-Philipson-Tooze.”

14 Weissmann et al. to Kendrew, June 7, 1974, in EMBO Archive, Box “Correspondence Kendrew-Philipson-Tooze.” In addition to Weissmann and Philipson, who materially drafted the letter, the other signatories were L. Crawford, F. Cuzin, R. Dulbecco, W. Fiers, B. Hirt, J. H. Subak-Sharpe, K. Weber, R. Weil, E. Winocour.

15 Kendrew to Weissmann, June 25, 1974, in EMBO Archive, box “Correspondence Kendrew-Philipson-Tooze.” The impact of the Weissmann-Philipson letter on the EMBL will be discussed in the following section.

16 Walter Bodmer, Hans Kornberg, Rodney Porter, Michel Stoker, John H. Subak-Sharpe, Maurice Wilkins.

17 Kendrew to Berg, July 19, 1974, in Paul Berg Papers, Department of Special Collections and University Archives, Stanford University Libraries, Stanford, California (hereafter Berg Papers), series 3, Box 13, folder 18.

18 Berg to Kendrew, August 13, 1974, in Berg Papers, series 3, Box 13, folder 18. The program was broadcast by BBC Two England on September 16, 1974, at 21.25. The description of the program in *Radio Times* referred to the “world ban” and asked the question: “Brave New World—now?” See BBC program index: https://genome.ch.bbc.co.uk/schedules/service_bbc_two_england/1974-09-16 (accessed on January 9, 2025).

19 Berg to Brenner, September 17, 1974, in Berg Papers, series 3, Box 13, folder 16.

20 Interview with Tooze by Weiner, March 26, 1976, pp. 40–41, in MIT-OHP, Box 14, folder 162. See Matthew Cobb’s contribution in this same *Journal of the History of Biology* Topical Collection.

21 Brenner to Berg, November 20, 1974, in Berg Papers, series 3, Box 13, folder 16.

22 “EMBC Fifth Ordinary Session (second part), Provisional Summary Record of the Meeting, December 12, 1974 (CEBM/74/8),” p. 11, in EMBO Archive, Box “EMBC-meetings.” The European Unit of Account (EUA) was a unit of account used in the European Communities from 1975 to 1979.

23 Interview with Kendrew by Weiner, March 25, 1976, p. 6, in Archive of Sir John Cowdery Kendrew, Oxford, Bodleian Libraries (hereafter, Kendrew Papers), MSS. Eng.c.2610.

24 Initially, Kendrew had also invited Dulbecco and Reichardt to join the EMBO delegation, but they did not accept. See on this, Tooze to Berg, December 16, 1974, in Berg Papers, series 3, Box 13, folder 20. The notion of “delegation” needs to be qualified. Berg didn’t want formal delegations. As Kendrew recalled it: “His [Berg] line was he didn’t want any official delegations from anybody, because it wasn’t that kind of a meeting. So I said well that’s fine, but if we pay the expenses of half a dozen people, whom we would agree between us (with him) were appropriate people, would he agree? And he was totally receptive to that:” Interview with Kendrew by Weiner, March 25, 1976, p. 4, in Kendrew Papers, MSS.Eng.c.2610.

25 “Minutes of the twenty-fourth Meeting of the EMBO Council, January 10, 1975,” p. 9, in EMBO Archive, box “EMBO Council meetings.”

26 Interview with Tooze by Weiner, March 26, 1976, p. 21, in MIT-OHP, Box 14, folder 162.

27 “EMBC Sixth Ordinary Session (first part), Report and Recommendations of the ad hoc committee on recombinant DNA molecules, established by the EMBO Council February 27, 1975 (CEBM/75/2);” annex 1 to the Report of the EMBO Delegation, in EMBO Archive, Box “EMBC-meetings.”

28 “EMBC Sixth Ordinary Session (first part), Report of the EMBO Delegation which attended the Conference on Recombinant DNA Molecules sponsored by the National Academy of Sciences—National Research Council of the United States of America, and held at Asilomar, California, 24–27 February 1975, July 3, 1975 (CEBM/75/2),” in EMBO Archive, box “EMBC-meetings.”

29 Delegates from the Netherlands and Ireland also took this position.

30 “EMBC Sixth Ordinary Session (first part), Provisionary Summary Record of the Meeting, July 3, 1975,” p. 16, in EMBO Archive, Box “EMBC meetings.”

31 “EMBC Sixth Ordinary Session (first part), Provisionary Summary Record of the Meeting, July 3, 1975,” p. 16, in EMBO Archive, Box “EMBC meetings.”

32 “EMBC Sixth Ordinary Session (first part), Provisionary Summary Record of the Meeting, July 3, 1975,” p. 21, in EMBO Archive, Box “EMBC meetings.”

33 On November 24 and December 15, 1975, Anderson organized a training course on the “safe handling of pathogens” at the Enteric Reference Laboratory in Colindale, London. The course was sponsored by EMBO and ESF, with funds coming from the European Economic Community. The participants attended in two batches of three: A. Bernardi (Paris), H. Pannekoek (Leiden), B. Niaudet (Paris) in November; C. Weissmann (Zurich), G. Magnusson (Stockholm), R. Streeck (Munich) in December. See “Minutes of the eighteenth Meeting of the Course Committee, 17 October 1975 (EMBO/CC/5/1975),” p. 7, in EMBO Archive, Box “EMBO Course Committee Documents, 1966–1977.”

34 “EMBC Sixth Ordinary Session (second part), November 26, 1975, Draft terms of reference of the EMBO Standing Advisory Committee on Recombinant DNA (CEBM/75/7),” in EMBO Archive, Box “EMBC-meetings.” To satisfy eventual requests concerning strains of bacteria, plasmids, and bacteriophages best suited for specific experiments, the EMBO SAC urged the creation of a type collection at EMBL or somewhere else in Europe at this meeting, and this also became a responsibility of the committee.

35 “Summary Record of the twenty-fifth Meeting of the EMBO Council, January 17, 1976,” p. 16, in EMBO Archive, Box “EMBO Council meetings.”

36 Tooze to all members of the EMBO Standing Advisory Committee on Recombinant DNA, December 17, 1975, in Cold Spring Harbor Laboratory Archives, Sydney Brenner Collection (hereafter Brenner Collection), series 4: Subject Files, subseries 1: General, Box 8, folder “EMBO Standing Advisory Committee on Recombinant DNA.”

37 “Summary Record of the First Meeting of the EMBO Standing Advisory Committee on Recombinant DNA, February 14–15, 1976 (final draft),” p. 2, in EMBO Archive, Box “Minutes EMBO Standing Advisory Committee.”

38 “Summary Record of the First Meeting of the EMBO Standing Advisory Committee on Recombinant DNA, 14–15 February 14–15, 1976 (second draft),” p. 4 (emphasis in the original), in EMBO Archive, Box “Minutes EMBO Standing Advisory Committee.” See also Norman (1976).

39 Within the EMBO SAC, Anderson, Murray, and Weissmann were in charge of this experimental analysis.

40 “Summary Record of the First Meeting of the EMBO Standing Advisory Committee on Recombinant DNA, February 14–15, 1976 (final draft), Appendix II,” in EMBO Archive, Box “Minutes EMBO Standing Advisory Committee.”

41 “Summary Record of the First Meeting of the EMBO Standing Advisory Committee on Recombinant DNA, February 14–15, 1976 (final draft),” p. 3, in EMBO Archive, Box “Minutes EMBO Standing Advisory Committee.”

42 Weissmann to Stetten, February 18, 1976, in EMBO Archive, Box “Minutes EMBO Standing Advisory Committee.”

43 In the conclusion of his 1977 book *The Ultimate Experiment: Man-Made Evolution*, which chronicles the rDNA controversy in the US, Wade celebrated the advent of a new eugenics era driven by genetic engineering (Wade 1977, p. 150).

44 Wade (1976, p. 236); see also Wright (1994, p. 186). During the Cambridge City Hall hearings on rDNA, in June–July 1976, Wade’s article was extensively quoted to denounce the external—European—influence on the contents of the NIH guidelines. We are grateful to Luis Campos for drawing our attention to this documentation.

45 The ESF ad hoc Committee on rDNA Research (Genetic Manipulation) was established in October 1975. It met three times in 1976 under the chairship of Povl Riis (University of Copenhagen). Tooze and Zachau were members.

46 Anderson and Brenner were members.

47 Murray and Tooze were members of COGENE, while Kendrew was ICSU’s President.

48 Tooze to all members of the EMBO Standing Advisory Committee on Recombinant DNA, April 20, 1976, in Brenner Collection, series 1: Correspondence, subseries 2: Institutional, Box 10, folder “EMBO.”

49 This was the Commission de Contrôle, established in 1975 under the auspices of the Délégation Générale à la Recherche Scientifique et Technique (DGRST).

50 Tooze to Flowers, June 9, 1978, in NIH, National Library of Medicine (Bethesda, MD), Archives and Modern Manuscripts Collection, Donald S. Fredrickson Papers, series 4 (“recombinant DNA materials”), Box 23 (“International, 1974–1984”), folder 8 (“EMBO 1978–1980”). We are grateful to Chris Donohue for drawing our attention to this archival material.

51 Rowe to Recombinant DNA Advisory Committee, April 14, 1978, in Brenner Collection, series 4: Subject Files, subseries 1: General, box 10, folder “GMAG, Jan–Jul 1978.” See also Wright 1994, pp. 298–99.

52 “EMBC Eleventh Ordinary Session (first part), Report of the Sixth Meeting of the EMBO Standing Advisory Committee on Recombinant DNA, February 17, 1980 (CEBM/80/2 E),” p. 4, in EMBO Archive, Box “EMBC-meetings.”

53 Murray and Tooze to Fredrickson, 21 December 1979, Appendix 1 to the “Report of the sixth Meeting of the EMBO Standing Advisory Committee on Recombinant DNA, February 17, 1980 (CEBM/80/2 E),” p. 4, in EMBO Archive, box “EMBC-meetings.”

54 “Report of the sixth Meeting of the EMBO Standing Advisory Committee on Recombinant DNA," February 17, 1980 (CEBM/80/2 E),” p. 4, in EMBO Archive, box “EMBC-meetings.” The EMBO SAC on rDNA welcomed the NIH decision to allow the cloning of DNA in *E. coli* K12 under P1 physical containment. With regard to the European context, the EMBO SAC was particularly worried about the stringency of the Dutch guidelines for rDNA experiments.

55 Starting from the third meeting of the EMBO SAC on rDNA, in May 1977, Brenner is not mentioned anymore as a member of the committee.

56 “Report of the sixth Meeting of the EMBO Standing Advisory Committee on Recombinant DNA," February 17, 1980 (CEBM/80/2 E),” p. 3, in EMBO Archive, box “EMBC-meetings.”

57 Weissmann et al. to Kendrew, June 7, 1974, in EMBO Archive, Box “Correspondence Kendrew-Philipson-Tooze.”

58 Kendrew to Weissmann, June 25, 1974, in EMBO Archive, Box “Correspondence Kendrew-Philipson-Tooze.”

59 Weissmann to Kendrew, July 8, 1974, in EMBO Archive, Box “Correspondence Kendrew-Philipson-Tooze.”

60 Kendrew to Weissmann, July 12, 1974, in EMBO Archive, Box “Correspondence Kendrew-Philipson-Tooze.”

61 Interview with Weissman by Weiner, May 29, 1975, pp. 27–28, in MIT-OHP, Box 14, folder 167.

62 Interview with Philipson by Weiner, July 18, 1977, pp. 44–45, in MIT-OHP, Box 12, folder 138.

63 Interview with Tooze by Weiner, March 26, 1976, pp. 18–19, in MIT-OHP, Box 14, folder 162.

64 “Draft Minutes of the First Ordinary Session (second part) of the EMBL Council, December 12-13, 1974 (EMBL/75/2),” p. 13, in Kendrew Papers, MSS.Eng.c.2524. The EMBL was legally separated from EMBO and EMBC. The EMBL Council included delegates and observers—both scientists (often EMBO Council members) and public servants—from the countries that had signed the intergovernmental agreement establishing the Laboratory on May 10, 1973: Austria, Denmark, France, Greece, Italy, Israel, the Netherlands, Norway, Spain, Sweden, Switzerland, United Kingdom, and West Germany.

65 “Draft Minutes of the First Ordinary Session (second part) of the EMBL Council, December 12–13, 1974 (EMBL/75/2),” p. 15, in Kendrew Papers, MSS.Eng.c.2524. Delegates included E. Andres and N. Roulet for Switzerland; W. Hutter and A. Rörsch for Netherlands; A. Alline and J. P. Ebel for France; Y. Saphir for Israel.

66 “Minutes of the twenty-fourth Meeting of the EMBO Council, January 10, 1975,” p. 10, in EMBO Archive, Box “EMBO Council meetings.”

67 “EMBC Sixth Ordinary Session (first part): Report of the EMBO delegation which attended the Conference on Recombinant DNA Molecules sponsored by the National Academy of Sciences—National Research Council of the United States of America, and held at Asilomar, California, 24–27 February 1975, July 3, 1975 (CEBM/75/2),” in Kendrew Papers, MSS.Eng.c.252.

68 “Draft Minutes of the Second Ordinary Session (first part) of the EMBL Council, July 3–4, 1975 (EMBL/75/11),” p. 18, in Kendrew Papers, MSS.Eng.c.2524.

69 “Draft Minutes of the Second Ordinary Session (first part) of the EMBL Council, July 3–4, 1975 (EMBL/75/11),” p. 18, in Kendrew Papers, MSS.Eng.c.2524.

70 “Draft Minutes of the Second Ordinary Session (first part) of the EMBL Council, July 3–4, 1975 (EMBL/75/11),” p. 19, in Kendrew Papers, MSS.Eng.c.2524.

71 “Draft Minutes of the Second Ordinary Session (first part) of the EMBL Council, July 3–4, 1975 (EMBL/75/11),” p. 21, in Kendrew Papers, MSS.Eng.c.2524.

72 Interview with Kendrew by Weiner, March 25, 1976, p. 20, in Kendrew Papers, MSS. Eng.c.2610.

73 “Draft Minutes of the Second Ordinary Session (first part) of the EMBL Council, July 3–4, 1975 (EMBL/75/11),” p. 22, in Kendrew Papers, MSS.Eng.c.2524.

74 J. Kendrew, “Proposal for an EMBL Facility for Genetic Engineering; Medium and High-Risk Containment Laboratories for Research on Recombinant DNA (EMBL/Fin.Com./75/29),” p. 1, in “Draft Minutes of the Second Ordinary Session (second part) of the EMBL Council, November 27, 1975 (EMBL/76/1),” Kendrew Papers, MSS.Eng.2524 (hereafter, Kendrew, “Proposal for an EMBL Facility for Genetic Engineering”).

75 Kendrew, “Proposal for an EMBL Facility for Genetic Engineering,” p. 1.

76 Kendrew, “Proposal for an EMBL Facility for Genetic Engineering,” p. 2.

77 “Draft Minutes of the Second Ordinary Session (second part) of the EMBL Council, November 27, 1975 (EMBL/76/1),” p. 14, in Kendrew Papers, MSS.Eng.2524.

78 “Resolution on the Genetic Engineering Program, November 27, 1975 (EMBL/75/33 E),” in Kendrew Papers, MSS.Eng.c.2525.

79 Other members of the EMBL Recombinant DNA Working Group were Giorgio Bernardi (Paris), Sydney Brenner (Cambridge), Hermann Bujard (Heidelberg), Reuben Leberman (Heidelberg), Heinz Schaller (head of the Institute of Microbiology at Heidelberg University), Vince Pirrotta (Basel), Hans G. Zachau (EMBL delegate of West Germany; member of several German and international committees on rDNA, including at the European Science Foundation and the International Council of Scientific Unions), and John Tooze. See Maaløe and Fasella, “Scientific Advisory Committee. Report of the Chairmen for the Period October 1974–December 1977, November 2, 1977 (EMBL/77/9), annex 3,” in Kendrew Papers, MSS.Eng.2527.

80 “Minutes of ninth meeting of the Scientific Advisory Committee of the EMBL*,* July 1–2, 1977 (EMBL/77/6),” p. 2, in Kendrew Papers, MSS.Eng.c.2527.

81 See for instance: G. R. Zelle Neuenheim,“'Alles harmlos.’ Genverschmelzungsforschung im EMBL,”*Kommunistische Volkszeitung*, January 30, 1978, 5; "Bisher noch keine Versuche in Stufe 4," *Heidelberger Nachrichten*, May 2, 1979, 5, in EMBL Archive, Heidelberg, fonds “COM-EMBL Strategy and Communication Materials, series B Press Clippings File 1 1971–1993,” Box 340, folder 1975–1987.

82 “Minutes of the tenth meeting of the Scientific Advisory Committee of the EMBL, November 25–26, 1977,” p. 3, in Kendrew Papers, MSS.Eng.c.2527.

83 “EMBL Annual Report 1978,” p. 36. EMBL Archive. https://archive.embl.org/index.php/annual-report-1978. Accessed 13 January 2025.

84 “Minutes of the twelfth meeting of the Scientific Advisory Committee of the EMBL*,* July 10–11, 1978 (EMBL/78/5),” p. 3, in Kendrew Papers, MSS.Eng.c.2528.

85 “Minutes of the tenth meeting of the Scientific Advisory Committee of the EMBL, November 25–26, 1977 (EMBL/78/2),” p. 4, in Kendrew Papers, MSS.Eng.c.2527.

86 “Minutes of the thirteenth meeting of the Scientific Advisory Committee of the EMBL, January 18–19, 1979 (EMBL/79/4),” p. 4, in Kendrew Papers, MSS.Eng.c.2530.

87 “Draft Minutes of the Seventh Ordinary Session (second part) of the EMBL Council, November 25, 1980 (EMBL/80/8),” p. 11, in Kendrew Papers, MSS.Eng.c.2531.

88 “Draft Minutes of the Fifth Ordinary Session (second part) of the EMBL Council, December 11 and 13, 1978 (EMBC/79/1),” pp. 14–18, in Kendrew Papers, MSS.Eng.c.2529.

89 “Draft Minutes of the Fourth Ordinary Session (first part) of the EMBL Council, July 6, 1977 (EMBL/77/4),” p. 10, in Kendrew Papers, MSS.Eng.c.2527. See also “EMBL Annual Report 1979,” pp. 46–47. EMBL Archive. https://archive.embl.org/index.php/annual-report-1979. Accessed 13 January 2025.

90 Kendrew to all delegates of the EMBL Council, November 17, 1978, in Kendrew Papers, MSS.Eng.c.2528.

91 Noreen Murray’s research group, in the Division of Cell Biology, was dedicated to the molecular genetics of procaryotes. See “EMBL Annual Report 1980,” pp. 28–29. EMBL Archive. https://archive.embl.org/index.php/annual-report-1980. Accessed 13 January 2025.

92 “Draft Minutes of the Sixth Ordinary Session (second part) of the EMBL Council, November 21, 1979 (EMBL/79/11),” p. 11, in Kendrew Papers, MSS.Eng.c.2530.

93 “Draft Minutes of the Seventh Ordinary Session (first part) of the EMBL Council, June 11, 1980 (EMBL/1980/3),” p. 12, in Kendrew Papers, MSS.Eng.c.2530.

94 “Draft Minutes of the Eighth Ordinary Session (first part) of the EMBL Council, June 3, 1981 (EMBL/1981/3),” p. 11, in Kendrew Papers, MSS.Eng.c.2531. Up to the end of 1979, research on rDNA at the EMBL had attracted in Heidelberg 12 postdoctoral fellows, 4 students and 13 visitors: see “EMBL Annual Report 1979,” p. 19. EMBL Archive. https://archive.embl.org/index.php/annual-report-1979. Accessed 13 January 2025.

95 “Message from Walter Scheel. Translation. Bonn, April 1978,” in Kendrew Papers, MSS.Eng.c.2468. The President sent this message for the EMBL inauguration, but he couldn’t attend in person as he was hosting a visit from Soviet leader Leonid Brezhnev.

96 “EMBL Annual Report 1976,” p. 3. EMBL Archive. https://archive.embl.org/index.php/annual-report-1976. Accessed 13 January 2025.

97 Interview with Tooze by Weiner, March 26, 1976, pp. 35–36, in MIT-OHP, Box 14, folder 162.

98 Interview with Tooze by Weiner, March 26, 1976, p. 85, in MIT-OHP, Box 14, folder 162.

99 Interview with Tooze by Weiner, March 26, 1976, p. 89, in MIT-OHP, Box 14, folder 162.

100 Interview with Tooze by Weiner, March 26, 1976, p. 88, in MIT-OHP, Box 14, folder 162.

101 “Summary Record of the twenty-fifth Meeting of the EMBO Council, January 17, 1976,” p. 5, in EMBO Archive, box “EMBO Council meetings.” The Committee included Nils Kjeldgaard, Arthur Rörsch, Paolo Fasella, Michael Sela, and John Tooze.

102 Tooze, *EMBC Retrospect and Prospect*, in EMBO Archive, Box “Future of EMBO.”

103 The EMBC Secretary General assisted the EMBC President and maintained continuity of business between EMBC sessions. He was an EMBO representative, and, in the exercise of his duties, he made use of EMBO services. See on this, Cassata 2024, pp. 35–37. Arthur Rörsch was the EMBC delegate for the Netherlands and EMBC Secretary General between 1975 and 1980.

104 “Summary Record of the twenty-sixth Meeting of the EMBO Council, January 8, 1977,” p. 6, in EMBO Archive, Box “EMBO Council meetings.”

105 “Summary Record of the twenty-sixth Meeting of the EMBO Council*,* January 8, 1977,” p. 6, in EMBO Archive, Box “EMBO Council meetings.”

106 “Summary Record of the twenty-sixth Meeting of the EMBO Council, January 8, 1977,” p. 7, in EMBO Archive, Box “EMBO Council meetings.”

107 “Summary Record of the twenty-seventh Meeting of the EMBO Council, September 24, 1977,” p. 9, in EMBO Archive, Box “EMBO Council meetings.”

108 “EMBC Ninth Ordinary Session (first part), Final draft version of the document prepared by the EMBO Council concerning the future of the General Programme beyond 1980, March 13, 1978, CEBM 78/2,” p. 9, in EMBO Archive, Box “EMBC-meetings”.

109 “EMBC Ninth Ordinary Session (first part), Final draft version of the document prepared by the EMBO Council of EMBO concerning the future of the General Programme beyond 1980, March 13, 1978, CEBM 78/2,” p. 16, in EMBO Archive, Box “EMBC-meetings.”

110 “EMBC Ninth Ordinary Session (second Part), Report of the Chairman of the Plenary Working Party established to consider the prolongation of the Agreement beyond April 2nd, 1980, October 13, 1978*,* CEBM/78/8,” pp. 7–8, in EMBO Archive, Box “EMBC-meetings.”

111 Tooze to Sela, October 13, 1978, in EMBO Archive, Box “EMBO Varia.”

112 “Summary Record of the thirty-first Meeting of the EMBO Council, January 26, 1980,” p. 9, in EMBO Archive, box “EMBO Council meetings.”

113 “EMBC Seventeenth Ordinary Session (first part), A Proposal Concerning a Prolongation of the EMBC Agreement beyond April 1988, 28 January 1986 (CEBM/86/1/E),” p. 3, in EMBO Archive, Box “EMBC-meetings.”

114 “EMBC Seventeenth Ordinary Session (first part), A Proposal Concerning a Prolongation of the EMBC Agreement beyond April 1988, 28 January 1986 (CEBM/86/1/E),”, p. 7, in EMBO Archive, Box “EMBC-meetings.”

115 OECD, *Recombinant DNA Safety Considerations* (Paris: OECD, 1986), p. 6. https://www.biosafety.be/sites/default/files/m00032689.pdf. Accessed 13 January 2025. The OECD was created out of the earlier Organization for European Economic Cooperation, which functioned as an ad-hoc agency assisting post-war reconstruction in Europe during the Marshall Plan. In the 1960s, the OECD played a crucial role in turning science policy into a regular part of government policies. Soon after the Asilomar Conference, the OECD Secretariat raised the question of rDNA as a potential topic for OECD action. See Gottweis (2005, pp. 331–332).

116 “EMBC Nineteenth Ordinary Session (first part), Provisional Summary Record of the Meeting, July 5, 1988, CEBM/88/8/E,” p. 18, in EMBO Archive, Box “EMBC-meetings.”

117 “EMBC Nineteenth Ordinary Session (first part), Provisional Summary Record of the Meeting, July 5, 1988, CEBM/88/8/E,” p. 19, in EMBO Archive, Box “EMBC-meetings.”

118 “Summary Record of the fortieth Meeting of the EMBO Council, October 1, 1988,” p. 23, in EMBO Archive, box “EMBO Council meetings.”

119 “EMBC Nineteenth Ordinary Session (second part), Provisional Summary Record of the Meeting, December 1, 1988, CEBM/88/14 E,” p. 19, in EMBO Archive, Box “EMBC-meetings.”

120 “EMBC Nineteenth Ordinary Session (second part), Provisional Summary Record of the Meeting, December 1, 1988, CEBM/88/14 E,” p. 19, in EMBO Archive, Box “EMBC-meetings.”

121 “EMBC Nineteenth Ordinary Session (second part), Provisional Summary Record of the Meeting, December 1, 1988, CEBM/88/14 E,” p. 19, in EMBO Archive, Box “EMBC-meetings.”

122 Franke to Jacques H. Weil (EMBC President), November 23, 1989, in “EMBC Twentieth Ordinary Session (second part), Provisional Summary Record of the Meeting, November 28, 1989, CEBM/89/16 E, Annex 1, p. 2,” in EMBO Archive, Box “EMBC-meetings.”

123 “EMBC Twentieth Ordinary Session (second part), Provisional Summary Record of the Meeting, November 28, 1989, CEBM/89/16 E,” p. 18, in EMBO Archive, Box “EMBC-meetings.”

124 “EMBC Twentieth Ordinary Session (second part), Provisional Summary Record of the Meeting, November 28, 1989, CEBM/89/16 E,” p. 18, in EMBO Archive, Box “EMBC-meetings.”

125 “Summary Record of the forty-seventh Meeting of the EMBO Council, September 23–24, 1995,” p. 28, in EMBO Archive, box “EMBO Council meetings.”

126 Gannon, “EMBO—The Future. A Discussion Document,” in “Summary Record of the forty-seventh Meeting of the EMBO Council, Annex 2, in EMBO Archive, box “EMBO Council meetings” (hereafter, “EMBO—The Future”).

127 Gannon, “EMBO—The Future,” p. 2.

128 Gannon, “EMBO—The Future,” p. 3. Italics added.

129 Gannon, “EMBO—The Future,” p. 4.

130 . See for example, *Molecular Biology in Society*, Heidelberg, 1997; and see *Molecular Medicine in Society*, Porto, 1998 and *Molecular Biology and the Environment*, Prague, 1999.

131 See, for instance, Gannon’s paper “Basic Research and Future Research Policy” (unpublished), approved by the EMBO Council at its 50th meeting in September 1998. See “Summary Record of the fiftieth Meeting of the EMBO Council, September 23–24, 1998,” p. 22, in EMBO Archive, Box “EMBO Council meetings.”

132 “Summary Record of the forty-fifth Meeting of the EMBO Council, September 27, 1993,” p. 8, in EMBO Archive, box “EMBO Council meetings.” The Committee was disbanded in 2001, when the EMBO Council decided to define “different combinations” of experts to consider “any future proposals” of peer-review: see “Summary Record of the fifty-fourth Meeting of the EMBO Council, September 25–26, 2001,” p. 34, in EMBO Archive, Box “EMBO Council meetings.”

133 “Summary Record of the forty-ninth Meeting of the EMBO Council, September 25–26, 1997,” p. 25, in EMBO Archive, Box “EMBO Council meetings.”

134 “Summary Record of the fifty-sixth Meeting of the EMBO Council, September 25–26, 2002,” p. 20, EMBO Archive, Box “EMBO Council meetings.”

135 Gannon, *EMBC/EMBO as a prototype a European Research Council* (abstract for the Danish conference, October 7–8, 2002), in EMBO Archive, Box “ERC Correspondence.”

136 “EMBC Thirty-third Ordinary Session (second part), Planning document on the future of the EMBC/EMBO; Building on a record of achievement, 23 October 2002,” CEBM/01/8 Rev 5 E, p. 24, in EMBO Archive, box “EMBC-meetings” (hereafter, “Planning document on the future of the EMBC/EMBO”).

137 In May 2002, Gannon proposed the implementation of an EMBO Research Award Programme, an EMBC Special Project conceived as a “pilot scale” or a “prelude” to ERC. See on this: Gannon, *A proposal to establish a Consortium of Foundations to launch an EMBO Research Award Programme*, in EMBO Archive, Box “ERC Correspondence.”

138 “Planning document on the future of the EMBC/EMBO,” p. 45.

139 “Planning document on the future of the EMBC/EMBO,” pp. 45–46.

140 “Planning document on the future of the EMBC/EMBO,” p. 2.

141 “EMBC Thirty-fifth Ordinary Session (first part), Formalisation of the process of an input on science policy to the EMBC from EMBO*,* 28 June 2004 (CEBM/04/19 E),” p. 2, in EMBO Archive, Box “EMBC meetings.” See also “EMBC Thirty-fifth Ordinary Session (first part), Report of the EMBC Strategic Working Party on the 8th meeting held in Heidelberg on 19 April 2004, CEBM/04/7 E,” pp. 8–9, in EMBO Archive, Box “EMBC meetings.” EMBC delegations had contested the use of the term “Academy” in July 2002, during the meeting of the 33rd EMBC Ordinary Session.

142 “EMBC Thirty-fifth Ordinary Session (first part), Formalisation of the process of an input on science policy to the EMBC from EMBO, 28 June 2004 (CEBM/04/19 E),” p. 3, in EMBO Archive, box “EMBC meetings.”

143 “EMBC Thirty-fifth Ordinary Session (first part), Provisional Summary Record of the Meeting, 28 June 2004 (CEBM/04/22 E),” p. 29, in EMBO Archive, box “EMBC meetings.”

144 “EMBC Thirty-fifth Ordinary Session (first part), Report of the EMBC Strategic Working Party on the 9th meeting held in Paris on 23 and 24 September 2004, CEBM/04/27 E,” pp. 8-9, in EMBO Archive, box “EMBC meetings.”

145 The collaboration and friendship between the two dated back to the mid-1960s, when Tooze spent two years (1965–1966) in Watson’s laboratory at Harvard University. Before accepting a position as a research administrator at the ICRF in 1970, Tooze had considered joining Watson at CSHL. The collaboration continued at a distance in the field of tumor virology. On this, see Morgan (2022, pp. 171–72). In February 1975, Watson and Tooze met at the Asilomar Conference while working on the updated edition of their “Tumor Viruses book” (Tooze 1973). See the correspondence between Watson and Tooze in CSHL Archives, James D. Watson Collection, RG II: Personal Papers series 2: Correspondence (hereafter JDW Collection), Box 76, folder “Correspondence Tooze, John.”

146 Tooze’s letter to contributors, undated, JDW Collection.

147 Interview with Tooze by Weiner, March 26, 1976, p. 57, in MIT-OHP, Box 14, folder 162.

## References

[CR1] Berg, Paul, David Baltimore, Herbert W. Boyer, Stanley N. Cohen, Ronald W. Davis, David S. Hogness, Daniel Nathans, et al. 1974. Potential biohazards of recombinant DNA molecules. *Science* 185: 303. 10.1126/science.185.4148.303. 4600381

[CR2] Campos, Luis A. 2024. A little care before we leap: The open letter that spurred the historic Asilomar conference turns 50. *Science* 385: 1424–1425. 10.1126/science.ads7339. 39325908 10.1126/science.ads7339

[CR3] Cantley, Mark F. 1995. The regulation of modern biotechnology: A historical and European perspective. In *Biotechnology. Legal, economic and ethical dimensions*, vol. 12, 2nd ed., ed. Dieter Brauer, 505–681. Weinheim: VCH.

[CR4] Cassata, Francesco. 2015. "A Cold Spring Harbor in Europe:" Euratom, UNESCO and the foundation of EMBO. *Journal of the History of Biology* 48: 539–573. 10.1007/s10739-015-9408-5. 25931208 10.1007/s10739-015-9408-5

[CR5] Cassata, Francesco. 2024. A "heavy hammer to crack a small nut?" The creation of the European molecular conference (EMBC), 1963–1970. *Annals of Science*. 10.1080/00033790.2024.2351511. 10.1080/00033790.2024.2351511 38752430

[CR6] de Chadarevian, Soraya. 2002. *Designs for life: Molecular biology after World War II*. Cambridge: Cambridge University Press.

[CR8] Fredrickson, Donald S. 1991. Asilomar and recombinant DNA: The end of the beginning. In *Biomedical politics*, ed. Kathi E. Hanna, 258–292. Washington: National Academy Press.

[CR32] Fried, Michael, Binie Klein, Kenneth Murray, Peter Greenaway, John Tooze, Werner Boll, and Charles Weissmann. 1979. Infectivity in mouse fibroblasts of polyoma DNA integrated into plasmid pBR322 or lambdoid phage DNA. *Nature* 279: 811–816. 10.1038/279811a0 10.1038/279811a0 221838

[CR9] Gottweis, Herbert. 1998. *Governing molecules: The discursive politics of genetic engineering in Europe and the United States*. Cambridge: MIT Press.

[CR10] Gottweis, Herbert. 2005. Transnationalizing recombinant-DNA regulation: Between Asilomar. EMBO, the OECD, and the European Community.* Science as Culture* 14: 325–338. 10.1080/09505430500369020. 10.1080/09505430500369020 16619468

[CR11] Jasanoff, Sheila. 2005. *Designs on nature: Science and democracy in Europe and the United States*. Princeton: Princeton University Press.

[CR12] Krige, John. 2002. The birth of EMBO and the difficult road to EMBL. *Studies in History and Philosophy of Biological and Biomedical Sciences C* 33: 547–564. 10.1016/S1369-8486(02)00013-4.

[CR13] Krige, John. 2003. The politics of European scientific collaboration. In *Companion to science in the twentieth century*, ed. John Krige and Dominique Pestre, 897–918. London: Routledge.

[CR14] Krimsky, Sheldon. 1982. *Genetic alchemy: The social history of the recombinant DNA controversy*. Cambridge: MIT Press.

[CR15] Loenen, Wil A.M., David T. F. Dryden, Elisabeth A. Raleigh, Geoffrey G. Wilson, and Noreen E. Murray. 2014. Highlights of the DNA cutters: A short history of the restriction enzymes. *Nucleic Acids Research* 42: 3–19. 10.1093/nar/gkt990. 24141096 10.1093/nar/gkt990 PMC3874209

[CR16] Mitzner, Veera. 2020. *European Union research policy: Contested origins*. Cham: Palgrave Macmillan.

[CR17] Morgan, Gregory J. 2022. *Cancer virus hunters: A history of tumor virology*. Baltimore: Johns Hopkins University Press.

[CR18] Norman, Colin. 1976. EMBO: Transatlantic translation. *Nature* 259: 617. 10.1038/259617a0.

[CR33] Philipson, Lennart. 1991. Turmoil in European biology. *Nature* 351: 91–92. 10.1038/351091a0. 10.1038/351091a0 2030737

[CR19] Scherwell, Chris. 1977. Europe: Harmony of practice. *Nature* 270: 94. 10.1038/270094a0. 11643420 10.1038/270094a0

[CR31] Schmeck Jr., Harold M. 1977. Rules on DNA studies viewed as too strict. *The New York Times*. December 18.

[CR20] Schneider, Friedrich. 1978. Genetic engineering guidelines. *Nature* 274: 10. 10.1038/274010a0.

[CR21] Singer, Maxine, and Dieter Söll. 1973. Guidelines for DNA hybrid molecules. *Science* 181: 1114. 10.1126/science.181.4105.1114.a. 11663279 10.1126/science.181.4105.1114

[CR22] Strasser, Bruno J. 2003. The transformation of the biological sciences in post-war Europe. *EMBO Reports* 4: 540–543. 10.1038/sj.embor.embor879. 12776168 10.1038/sj.embor.embor879 PMC1319215

[CR30] Tooze, John. 1973. *The molecular biology of tumor viruses*. New York: Cold Spring Harbor Laboratory Press.

[CR23] Tooze, John. 1978. Harmonizing guidelines: Theory and practice. In *Genetic engineering*, ed. Herbert W. Boyer and Simonetta Nicosia, 279–285. Amsterdam: Elsevier.

[CR24] Wade, Nicholas. 1976. Recombinant DNA: The last look before the leap. *Science* 192: 236–238. 10.1126/science.11643306. 11643306 10.1126/science.11643306

[CR25] Wade, Nicholas. 1977. *The ultimate experiment: Man-made evolution*. New York: Walter and Company.

[CR26] Walgate, Robert. 1978. Genetic engineering guidelines: Europe hopeful about collaboration. *Nature* 273: 331. 10.1038/273331b0.

[CR27] Watson, James D. 1977. In defense of DNA. *The New Republic* 176: 11–14. 11661812

[CR28] Watson, James D., and John Tooze. 1981. *The DNA story. A documentary history of gene cloning*. San Francisco: W. H Freeman and Company.

[CR29] Wright, Susan. 1994. *Molecular politics: Developing American and British regulatory policy for genetic engineering, 1972–1982*. Chicago: University of Chicago Press.

